# Histamine deficiency aggravates cardiac injury through miR-206/216b-Atg13 axis-mediated autophagic-dependant apoptosis

**DOI:** 10.1038/s41419-018-0723-6

**Published:** 2018-06-07

**Authors:** Suling Ding, Mieradilijiang Abudupataer, Zheliang Zhou, Jinmiao Chen, Hui Li, Lili Xu, Weiwei Zhang, Shuning Zhang, Yunzeng Zou, Tao Hong, Timothy C. Wang, Xiangdong Yang, Junbo Ge

**Affiliations:** 1 Shanghai Institute of Cardiovascular Diseases, Zhongshan Hospital, Institutes of Biomedical Sciences, Fudan University, Shanghai, 200032 China; 2 Department of Cardiac Surgery, Zhongshan Hospital, Fudan University, Shanghai, 200032 China; 3 Department of Cardiology, Zhongshan Hospital, Fudan University, Shanghai, 200032 China; 4 Department of Medicine and Irving Cancer Research Center, Columbia University, New York, NY 10032 USA

## Abstract

Histamine is a widely distributed biogenic amine involved in the regulation of an array of biological processes. Serum histamine level is markedly elevated in the early stages of acute myocardial infarction, whereas the role it plays remains unclear. Histidine decarboxylase (HDC) is the unique enzyme responsible for histamine production, and cardiac injury is significantly aggravated in HDC knockout mice (HDC^−/−^), in which histamine is deficient. We also observed that autophagy was highly activated in cardiomyocytes of HDC^−/−^ mice post acute myocardial infarction (AMI), which was abolished by compensation of exogenous histamine. The in vivo and in vitro results showed that acting through histamine 1 receptor, histamine increased miR-206 and miR-216b, which worked in concert to target to Atg13, resulting in the reduction of autophagy activation under hypoxia and AMI condition. Further study revealed that Atg13 interacted with FADD to promote the activation of caspase-8 and cell apoptosis. Taken together, these data unveil a novel intracellular signaling pathway involved in histamine regulating myocardial autophagy and apoptosis under hypoxia and AMI condition, which might help to more comprehensively evaluate the usage of histamine receptor antagonists and to develop new therapeutic targets for myocardial infarction.

## Introduction

Myocardial infarction (MI) involves multiple pathological processes that are initiated by ischemia-induced myocardial injury, followed by leukocytes recruitment, tissue repair, and myocardial remodeling^1, 2^. In the past decades, treatment of patients with acute MI (AMI) has been significantly improved owing to the application of intracoronary angioplasty and stent. However, more patients subsequently suffer from left ventricular dysfunction and heart failure^3^. Cardiac necrosis and apoptosis are both dead forms of myocyte loss, which leads to fibrosis repair and heart failure after MI^4^. Further understanding of the mechanism of cardiomyocyte loss may offer new therapeutic tactics for AMI patients.

Apoptosis is an energy-requiring process of programmed cell death that is defined by the activation of cysteine proteases known as caspases^5, 6^. Apoptosis plays an important role in various pathophysiological processes^7, 8^, and significantly contributes to myocyte cell death in MI^8, 9^. Patients who developed heart failure after AMI are associated with significantly increased apoptotic rates^4^.

Macroautophagy (here referred to as autophagy) is an intracellular degradation system by which cytoplasmic components are degraded in lysosomes^10, 11^. In the process of autophagy, an isolation membrane sequesters part of the cytoplasm to form an autophagosome, which subsequently fuses with lysosomes to enzymatically degrade sequestered materials. Although autophagy occurs at low levels under normal conditions, it is highly activated during ischemic heart disease^12, 13^. Autophagy was shown as an adaptive response to protect myocardium from acute ischemic death^13–15^, whereas it is also reported to exacerbate cardiomyocyte death and heart failure^15–17^. The functional role of autophagy in cardiovascular diseases is still poorly understood^17, 18^.

The crosstalk between apoptosis and autophagy is common and complicated^19, 20^. Several molecules required for autophagy also play key roles in regulating apoptosis^21, 22^. For example, the essential autophagy-related protein Beclin 1 is cleaved by caspases and stimulate the mitochondrial pathway of apoptosis^21^. Although the relationship between autophagy and apoptosis has been acknowledged^23^, the underlying regulatory mechanisms remain unclear.

Histamine is a biogenic amine, which is widely distributed and possesses many biological functions^24–27^. There are growing researches including ours reporting that histamine in the serum and myocardium increased significantly after AMI^26, 28–31^. Recent work demonstrated that histamine deficiency exacerbates AMI-induced cardiomyocyte apoptosis, indicating that endogenous histamine might exhibit a protective effect during MI^30^. Similar results were also found in cerebral ischemia that histamine alleviates neuronal damage^32–34^. However, the underlying mechanisms need more investigation.

This study reports that histamine exhibits a protective effect on AMI-induced cardiomyocytes apoptosis by repressing overactivated cardiac autophagy. Mechanically, histamine upregulates the expression of miR-206 and miR-216b via histamine 1 receptor, which act together to downregulate autophagy-related gene 13 (Atg13) expression, leading to the diminution of autophagy activity and caspase-8 activation.

## Methods

### Patients’ characteristics and serum histamine determination

Serum was taken from patients who were admitted to our hospital (Zhongshan Hospital, Fudan University). All of these patients claimed to have symptoms, including chest pain, chest tightness, palpitation, etc. Angiography was conducted to determine the diagnosis of acute myocardial infarction (AMI). Patients with confirmed AMI (1–3 day from onset) were selected. Healthy individuals confirmed by physical examination were randomly selected as controls and matched to cases based on age and gender. Serum concentration of histamine was determined using Histamine ELISA Kit (EA213/96, Eagle Biosciences) and detected by a microplate reader (SpectraMax M5). The investigations complied with the ethical guidelines of the 1975 Declaration of Helsinki and were approved by the review boards on human subject research in our institution (Zhongshan Hospital, Fudan University). Informed consent was obtained from all participants.

### Reagents

Histamine dyhydrochloride (C5H9N3.2HCL), 3-MA (3-methyl-3H-purin-6-amine), Z-VAD-fmk, Z-IETD-fmk, pyrilamine, cimetidine, and bafilomycin A1 were obtained from Sigma. Antibodies were obtained from the following sources: rabbit polyclonal anti-LC3 (Abcam), Rabbit polyclonal p62 antibody (Abcam), rabbit polyclonal anti-Histamine Receptor 1 (ABGENT), rabbit polyclonal anti-Histamine Receptor 2 (ABGENT), rabbit polyclonal anti-cleaved caspase-3 (Cell Signaling, 9661), mouse monoclonal anti-caspase-8 (Cell Signaling), rabbit polyclonal anti-Atg13 (Abcam), mouse monoclonal anti-FADD (Abcam), rabbit monoclonal anti-Beclin 1 (Abcam).

### Cell culture and treatment

H9c2 cardiomyocytes, a fetal cardiomyocyte-derived cell line (American Type Culture Collection) were cultured in Dulbecco’s Modified Eagle Medium (DMEM) supplemented with 10% fetal bovine serum and 100 μg/ml penicillin/streptomycin. HEK293 was cultured in DMEM supplemented with 10% fetal bovine serum and 100 μg/ml penicillin/streptomycin. Histamine (10^−6^ M, 10^−5^ M, 10^−4^ M), H1R antagonist (1 μM pyrilamine) and H2R antagonist (50 μM cimetidine) were added to treat cells. For hypoxia, cells were maintained in a hypoxic chamber (Billups Rothenberg, MIC-101, Del Mar, California) flushed with a preanalyzed gas mixture of 1% O_2_, 5% CO_2_, and 95% N. For transfection, cells were plated 24 h before transfection, and the indicated miRNAs mmics, antagomirs, or plasmids were transfected using Lipofectamine 2000 according to the manufacturer’s protocol.

### Quantitative reverse transcription-polymerase chain reaction

Stem-loop quantitative reverse transcription-polymerase chain reaction (qRT-PCR) for mature miRNA was performed on an Applied Biosystems ABI Prism 7000 sequence detection system. Total RNA was extracted using Trizol reagent. After DNAse I (Takara, Japan) treatment, RNA was reverse transcribed with reverse transcriptase (ReverTra Ace, Toyobo). The results of qRT-PCR were normalized to that of U6. The sequences of U6 primers were forward: 5′-GCTTCGGCAGCACATATACTAA-3′; reverse: 5′-AACGCTTCACGAATTTGCGT-3′. qRT-PCR analysis for histidine decarboxylase (HDC), H1R, H2R, H3R, and H4R were performed and standardized to control values of glyceraldehyde 3-phosphate dehydrogenase (GAPDH). Human HDC forward primer: 5′-GGACCCAGTTGATGACTGCT-3′; reverse: 5′-AGCGCACCGTCTTCTTCTTA-3′. Human GAPDH forward primer: 5′-CCTGCACCACCAACTGCTTA-3′; reverse: 5′-GGCCATCCACAGTCTTCTGG-3′. Mouse HDC forward primer: 5′ -TTAGTCTTTGGGTGTTCCTGGTCA-3′; reverse: 5′-CCCTGTTGCTTGTCTTCCTCAATA-3′; Mouse GAPDH forward primer: 5′ -GACATCAAGAAGGTGGTGAAGCAG-3′; reverse: 5′ -ATACCAGGAAATGAGCTTGACAAA-3′. Rat H1R forward primer: 5′-CCGGACCACAGACTCAGACA-3′; reverse: 5′-GAGTGTGAGCGGAGCCTCTT-3′. Rat H2R forward primer: 5′-GTGTACGGACTGGTGGATGG-3′; reverse: 5′-CCAGGAGCTGATGTGGTTGA-3′. Rat H3R forward primer: 5′-AGCTCGTTCTCACGTGTCCA-3′; reverse: 5′-TAGACGCAGCAGCTCTCAGC-3′. Rat H4R forward primer: 5′-AAGCAACCAGCACCTTCACA-3′; reverse: 5′-GCTCGAGTGGACAGAGCAAG-3′. Rat GAPDH forward primer: 5′-CCTGCACCACCAACTGCTTA-3′; reverse: 5′-GGCCATCCACAGTCTTCTGA-3′. A standard curve was calculated using the linear range of the dilution series (10 fmol down to 10 amol) and used for absolute quantification of the respective miRNAs.

### Immunoprecipitation

Cells were washed twice with cold phosphate-buffered saline (PBS), lysed with ice-cold lysis buffer, incubated for 30 min on ice, and centrifuged for 10 min at 4 °C. Immunoprecipitation was performed using Atg13 antibody or immunoglobulin G antibody as negative control, and the immune complexes were captured with protein A-agarose beads (Amersham Biosciences, Piscataway, NJ, USA). After three washes with cell lysis buffer, bead-bound proteins were subjected to sodium dodecyl sulfate-polyacrylamide gel electrophoresis (SDS-PAGE) and analyzed by western blot analysis.

### Immunoblotting

H1R, H2R, ATG13, LC3, p62, caspase-8, cleaved caspase-3, and FADD protein content were determined in lysates that had been treated as the indicated procedure. In brief, the harvested cells were washed once with cold PBS and re-suspended for 20 min on ice in a lysis buffer containing 20 mM Tris-HCl (pH 7.5), 0.5% Nonidet P-40, 0.5 mM phenylmethylsulfonyl fluoride, and 0.5% protease inhibitor cocktail (Sigma). The high-speed supernatant (10000 × g) was collected and proteins (30 μg) were separated by SDS-PAGE and transferred onto nitrocellulose membranes. Membranes were blocked in 5% non-fat dry milk in Tris-buffered saline-Tween 1% (TBS; 0.05 M Trizma base, 0.9% NaCl, and 1% Tween-20) and incubated overnight with the primary antibodies at 4 °C. The membranes were incubated at room temperature for 1 h with the relevant secondary antibodies conjugated to horseradish peroxidase and blots were developed by enhanced chemiluminescence detection (Amersham-Pharmacia Biotech).

### TUNEL assay

Cells were fixed in 4% paraformaldehyde in PBS and permeabilized with 0.1% Triton X-100 in 0.1% sodium citrate. An in situ apoptotic cell death detection kit (Fluorescein, Roche Applied Bio Sciences) based on terminal deoxynucleotidyl transferase dUTP nick end labeling (TUNEL) assay was used as per manufacturer’s instruction to detect apoptotic cells. Negative controls were included in each case by omitting TUNEL enzyme terminal deoxynucleotidyl transferase reaction mixture and incubating the cells with the label solution. PBS containing 5 μg/ml 4′,6′-diamidino-2-phenylindole (Vector Laboratories) was prepared to stain nuclei. Sections were examined with a Zeiss LSM510 META microscope. The percentage of apoptotic nuclei was calculated. A total of 100–150 cells were counted in 20–30 random fields. For apoptosis analysis by TUNEL assay in heart sections, the procedure is the same except for that cardiomyocytes were stained with the α-actinin antibody (A7811, Sigma). An investigator blind to the treatment quantified 20 random fields of samples.

### Confocal imaging

Cells were grown overnight on coverslips in 24-well plate. After treatment, cells were washed with PBS, fixed in 4% paraformaldehyde, and permeabilized with 0.1% Triton X-100. The fluorescent images were analyzed using an Olympus-FV500 multi-laser confocal microscope. The number of cells with GFP-LC3 punctuate structures was determined for a minimum of 100 cells.

### Preparation of RNAi construct of Atg13 and FADD

Small interfering RNA (siRNA) oligonucleotides specific for Atg13 and FADD were designed using the Ambion’s siRNA design tool (http://www.ambion.com/techlib/misc/siRNA_finder.html) and purchased from GenePharma Co. Ltd. Atg13 RNAi (Atg13-Si) sense sequence is 5′-GGAAAUUUGGUGUCUUGAATdT-3′; Atg13-Si antisense sequence is 5′-UUCAAGACACCAAAUUUCCTdT-3′. The Atg13-siRNA negative control (scramble) sense sequence is 5′-AGUCUAGUAUGUGUAGAGUTdT-3′; scramble antisense sequence is 5′-ACUCUACACAUACUAGACUTdT-3′. FADD RNAi (FADD-Si) sense sequence is 5′-GCGGGUGGCAUUUGACAUUTdT-3′; FADD-Si antisense sequence is 5′-AAUGUCAAAUGCCACCCGCTdT-3′. The FADD-Si negative control (scramble) sense sequence is 5′-AUCAUGCUAUGCGUGGUGGTdT-3′; scramble antisense sequence is 5′-CCACCACGCAUAGCAUGAUTdT-3′. Transfection of siRNAs was performed using Lipofectamin^TM^ 2000 according to the manufacturer’s instructions.

### Transmission electron microscopy

Conventional electron microscopy was performed as described previously^35^. In brief, cells were fixed with 2.5% glutaraldehyde and then postfixed with 1% osmium tetraoxide, dehydrated in a graded series of ethanol concentrations and embedded in Embed812 resin. The ultrathin sections were mounted on copper grids and then double-stained with uranyl acetate and lead citrate. The number of autophagic vacuoles was determined for a minimum of 100 cells. Heart ultrastructural analysis was also performed. The samples were examined and photographed with a FEI Tecnai spirit transmission electron microscope.

### Adenoviral constructions and infection

Atg13 cDNA were from sino biological. The GPF-LC3 expression plasmid was from Cell Q5 Biolabs Inc. The adenoviruses harboring Atg13 were constructed using the Adeno-X expression system (Clontech). Viruses were amplified in HEK293 cells. Cells were infected with the viruses at the indicated multiplicity of infection (moi) for 1 h. After washing with PBS, the culture medium was added and cells were cultured until the indicated time. Beclin 1 siRNA sequence is 5′-GATCCTGGACCGGGTCACC-3′. ATG13-siRNA sequence is 5′-GGAAAUUUGGUGUCUUGAA-3′. They were cloned into Q6 pSilencer adeno 1.0-CMV vector (Ambion) according to the manufacturer’s instructions.

### Preparation of the luciferase construct of Atg13 3′-UTR and luciferase assay

Atg13 3′-UTR was amplified by PCR with primer sets: 5′-CTCTTCCTCAGCTGCTTCCTAGC-3′ and 5′-GAGAGCATCCTTCCATTGTGTAG-3′. Mutations were generated with QuikChange II XL Site-Directed Mutagenesis Kit (Stratagene) and sequence confirmed. Wildtype and mutated 3′-UTRs were subcloned into the pGL3 vector (Promega) immediately downstream of the stop codon of the luciferase gene. For luciferase assay, cells in 24-well plates were co-transfected with the plasmid constructs of 200 ng/per well of empty pGL3, pGL3 harboring the wildtype 3′-UTR (Atg13-WT-3′ UTR) or the mutated 3′-UTR (Atg13-MUT1-3′ UTR, Atg13-MUT2-3′ UTR, Atg13-MUT1/2–3′ UTR) of Atg13, along with 45 nmol/L miR-206 and 35 nmol/L miR-216b using Lipofectamine 2000 (Invitrogen). pRL-TK vector containing *Renilla* luciferase cDNA served as the internal control. Mimic control (mimic-NC) served as a negative control. After 36 h of transfection, cells were lysed and luciferase activity was measured with the dual luciferase kit (Promega) according to the manufacturer’s instruction.

### Myocardial infarction model

HDC knockout (HDC^−/−^) mice were generously provided by Professor Timothy C. Wang from Columbia University. The generation of HDC-EGFP and HDC^−/−^ mice has been described in previous papers^30, 36^. Balb/C mice and C57BL/6 mice were purchased from the Department of Laboratory Animal Science, Fudan University, to serve as background controls. All mice were housed under specific pathogen-free conditions in an animal room with a 12/12 h day/night cycle with free access to water and food. This study was performed in strict accordance with the recommendations from the Guide for Animal Management Rules from the Ministry of Health of the People’s Republic of China. The protocol was approved by the Committee on the Ethics of Animal Experiments of Fudan University (approval reference number: SY2014.2.001.002). Surgery to induce myocardial infarction was performed in mice as described previously^30^. In brief, mice were anesthetized by inhalation of isoflurane, were intubated with a 22-G intravenous catheter, and then were fully anesthetized with 1.0–2.0% isoflurane gas while being mechanically ventilated on a positive pressure ventilator. Left thoracotomy was performed at the fourth intercostal space, and the pericardium was stripped to expose the heart. The left descending coronary artery was identified and occluded with an 8–0 silk ligature that was placed around it. The success of the ligation was confirmed when the anterior wall of the left ventricle turned pale. The chest cavity was closed, and the animal was placed in a cage on a heating pad. Sham-operated mice underwent the same surgical procedures except that the suture placed under the left anterior descending artery was not tied. From 3 days before surgery, histamine (1 mg/kg/d or 4 mg/kg/d), H1R antagonist (pyrilamine, 10 mg/kg/d), H2R antagonist (cimetidine, 10 mg/kg/d), bafilomycin A1 (0.3 mg/kg/d), and Z-IETD-fmk (5 mg/kg/d) were administered intraperitoneally daily until euthanasia. For intracoronary delivery of Beclin 1 siRNA (Ad-si-Beclin) or Atg13-siRNA (Ad-si-Atg13) adenoviruses, mice were anesthetized. The chest was then opened and 2 × 10^10^ moi adenoviruses were injected with a catheter from the apex of the left ventricle into the aortic root while the aorta and pulmonary arteries were cross-clamped. The clamp was maintained for 20 s when the heart pumped against a closed system. The chest was then closed and the mice were returned back to cage for recovery. We used chemically modified antisense oligonucleotides (Antagomir, Anta) to inhibit miR-206 and miR-216b expression. We intravenously injected the mice three consecutive days with antagomir (Anta) and antagomir negative control (Anta-NC) at doses of 10 mg/kg body weight, as well as mimics or mimics negative control (mimic-NC) at doses of 13.5 mg/kg body weight before MI surgery until euthanasia.

### Echocardiographic assessment

Transthoracic echocardiographic analysis was performed on mice after the sham or MI surgery as we described. Echocardiographic parameters such as systolic left ventricular internal diameters and diastolic left ventricular internal diameters (LVIDd) were measured. Fractional shortening of left ventricular diameter was calculated as [(LVIDd – LVIDs)/LVIDd] × 100. After in vivo evaluation of cardiac function the mice were killed and the hearts were harvested, weighted, and used for next experiments.

### Infarct size assessment

After 3 days of ischemia, the infarct size was determined with triphenylte trazoliumchloride (TTC) staining. In brief, the mice were killed and the hearts were removed and sliced into five 1.0-mm thick sections perpendicular to the long axis. The sections were then incubated with 1% TTC (Sigma) in phosphate solution at 37 °C for 10 min. The areas of infarcted tissue (TTC-negative staining area) and the whole left ventricle were determined by computer morphometry using Image-Pro Plus 6.0 software.

### Statistical analysis

Data are expressed as the mean ± s.e.m. of at least three independent experiments for each cellular experimental group and at least five independent experiments for each animal group. We evaluated the data with Student’s *t* test. We used a one-way analysis of variance for multiple comparisons. A value of *P* < 0.05 was considered significant.

## Results

### Histamine levels increased in MI subjects and histamine deficiency aggravated myocardial apoptosis in AMI mice

**Fig. 1:**
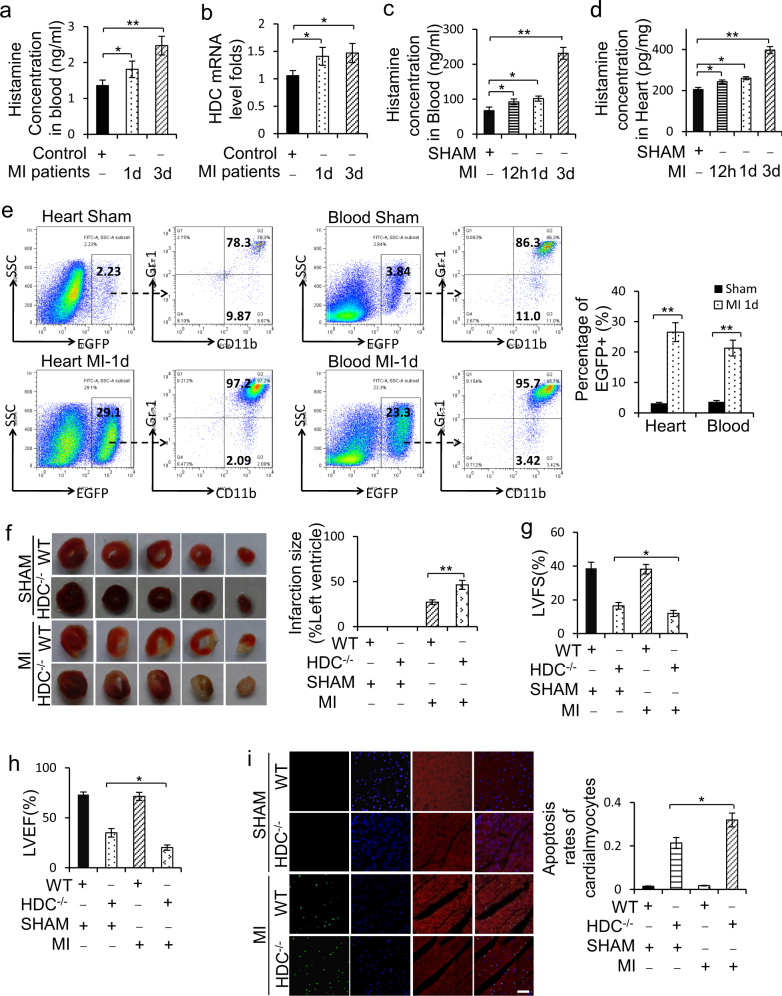
Histamine level increases and histamine deficiency aggravates myocardial apoptosis post MI. **a** Histamine concentration in the serum from AMI patients at 1d and 3d from onset and healthy people group was determined by Elisa kit (*n* = 15 in patients at 1d, *n* = 8 in patients at 3d, *n* = 15 in healthy control group). **b** HDC mRNA level in blood mononuclear cells of AMI patients at 1d and 3d from onset and healthy controls was analyzed by qRT-PCR (*n* = 15 in patients at 1d, *n* = 8 in patients at 3d, *n* = 15 in healthy controls group). **c**, **d** Histamine in the serum **(c**) and in the cardiac tissue (**d**) of AMI murine model at indicated time after AMI surgery was determined by Elisa kit (*n* = 5 for the sham group and *n* = 8 for each of MI groups). h, hour; d, day. **e** The percentage of EGFP^+^ myeloid cells in the infarcted myocardium and peripheral blood of HDC-EGFP mice at 1d post MI was analyzed by FACS (*n* = 5 for the sham group and *n* = 8 for each of MI groups). **f** Representative myocardial cross-sections of TTC-stained hearts. Red-colored regions indicate non-ischemic area, and pale-colored regions indicate ischemic area. Infarct size was analyzed at 3d after AMI surgery (*n* = 5 for the sham group and *n* = 5 for each of MI groups). **g**, **h** Echocardiographic analysis of cardiac function 1 week after AMI surgery. LVEF, left ventricular eject fraction. LVFS: left ventricular fraction shortening. *n* = 5 for the sham group and *n* = 8 for each of the MI groups. **i** Myocardial cell apoptosis was analyzed by TUNEL assay. TUNEL-positive nuclei (apoptotic cells) are green. Nuclei stained by DAPI show blue. Cardiomyocytes were labeled with a-actinin. scale bar, 50 μm. *n* = 3 for the sham group and *n* = 5 for each of MI groups. Results are representative of three independent experiments. *P < 0.05; **P < 0.01

Histamine levels were determined in serum samples collected from patients with angiographically confirmed MI and healthy control people. The result of enzyme-linked immunosorbent assay showed that histamine level was significantly elevated in the serum of MI patients compared with control people at 1 day and 3 days after MI (Fig. 1a). The mRNA level of HDC, which is the unique enzyme for the production of endogenous histamine by decarboxylation of L-histidine^36^, was upregulated in mononuclear cells isolated from the blood of MI patients compared with controls (Fig. 1b). Similar to the findings in AMI patients, histamine was consistently increased in serum and cardiac tissue of wildtype (WT) mice at 12 h, 1 day, and 3 days post MI (Fig. 1c, d). HDC mRNA level was likewise elevated in blood mononuclear cells from WT mice after MI (Supplementary Figure 1a). Furthermore, we identified a large number of HDC-expressing cells in the infarcted hearts and peripheral blood of HDC-EGFP transgenic mice at 1 day post MI (Fig. 1e). These EGFP^+^ cells were primarily defined as CD11b^+^ and Gr-1^+^ myeloid cells by flow cytometry (FACS) (Fig. 1e). Furthermore, FACS data demonstrated that EGFP^+^ are mainly expressed in CD11b^+^Gr-1^+^ and CD11b^+^Ly6C^+^ subsets but not in CD11b^+^Ly6C^high^ M1-type macrophages in the hearts of MI mice (Fig. 1e; Supplementary Figure 1b). Thus, the increase of histamine in the hearts of MI mice might be released from HDC-expressing CD11b^+^ myeloid cells recruited from the peripheral blood.

To explore the role of histamine in the pathogenesis of MI, HDC knockout mice (HDC^−/−^) were applied to the following studies, in which histamine level was undetectable in the myocardium before and after MI surgery (data not shown). The result of TTC staining showed larger infarct size in the hearts of HDC^−/−^ mice than in WT at 3 days after MI (Fig. 1f). Echocardiography studies confirmed a significantly reduced left ventricular ejection fraction (LVEF) and left ventricular fractional shortening in HDC^−/−^ mice than WT mice at 7 days post MI (Fig. 1g, h). In addition, TUNEL assay showed that histamine deficiency increased cardiomyocytes apoptosis in HDC^−/−^ mice compared with WT mice at 3 days after MI (Fig. 1i).

### Histamine represses AMI-induced autophagy and cell death in cardiomyocytes

**Fig. 2:**
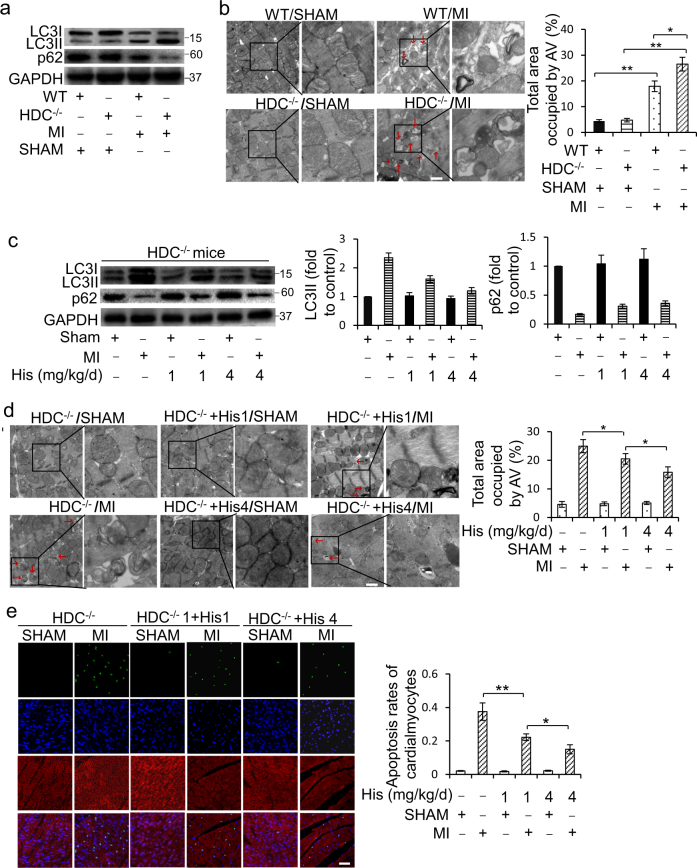
Histamine suppresses cardiomyocytes autophagy overactivation and cell death in AMI HDC^−/−^ mice. **a** Western blot analysis of the autophagy-related proteins LC3 and p62 in the hearts at 3d post AMI. **b** Representative electron micrographs (EM) of hearts from HDC^−/−^ mice and WT mice at 3d post AMI. Bar = 1 μm. Quantification of autophagic vacuoles (red arrows) is shown in the right panel (*n* = 3 for the sham group and *n* = 5 for each of MI groups). **c** HDC^−/−^ mice were administered exogenous histamine intraperitoneally for 3 consecutive days at the dose of 1 mg/kg/d or 4 mg/kg/d before exposed to AMI surgery. The levels of LC3 and p62 in hearts were analyzed by immunoblot at 3d post AMI (*n* = 5 for each group). **d** HDC^−/−^ mice were treated as **c**. Representative electron micrographs of hearts at 3d post AMI surgery. Bar = 1 μm. Quantification of autophagic vacuoles (red arrows) is shown in the right panel. *n* = 3 for the sham group and *n* = 5 for each of MI groups. **e** HDC^−/−^ mice were treated as **c**. Apoptosis was analyzed by TUNEL assay at 3d after surgery. TUNEL-positive nuclei (apoptotic cells) are green. Nuclei stained by DAPI show blue. Cardiomyocytes were labeled with a-actinin. Scale bar, 50 μm. *n* = 3 for the sham group and *n* = 5 for each of MI groups. *P < 0.05; **P < 0.01

Given that autophagy is closely related to apoptosis in myocardial ischemia, we proposed that histamine deficiency might have an effect on cardiomyocyte autophagy. Autophagy was activated in the infarcted hearts revealed by an augmentation in LC3II levels in WT mice after MI (Supplementary Figure 2a). However, LC3II levels were significantly higher in the hearts of HDC^−/−^ mice than that in WT mice post MI (Fig. 2a; Supplementary Figure 2b). As a portion of LC3II is degraded by autophagosome fusion with lysosomes, to determine whether the accumulation of LC3II was owing to an increase in autophagy induction or a decrease in autophagic degradation, p62, a selective substrate of autophagic degradation, was measured. In HDC^−/−^ mice, the ischemia-induced decrease in p62 level was much more obvious than in WT mice (Fig. 2a). The electron micrographic (EM) analysis of double membrane bound autophagic vesicles showed that autophagy was activated both in WT and HDC^−/−^ mice post MI, but the accumulation of autophagic vesicles was much more intensive in HDC^−/−^ than in WT mice (Fig. 2b). To confirm the effect of histamine on cardiomyocytes autophagy after MI, exogenous histamine was administrated into HDC^−/−^ mice for 3 consecutive days pre-MI, increasing the levels of histamine in the serum and hearts of HDC^−/−^ mice (Supplementary Figure 2c and 2d). In the histamine-treated HDC^−/−^ mice, the increase of LC3II formation and p62 degradation induced by MI were countered (Fig. 2c). So did the accumulation of autophagic vesicles (Fig. 2d). Bafilomycin A1 (BFA) is a lysosomal inhibitor that blocks autophagosome–lysosome fusion to prevent the final digestion step in autophagic process^37^. Our results showed that histamine reduced LC3II formation and autophagic vesicles accumulation even in the presence of BFA (Supplementary Figure 2e and 2f). In addition, enforced histamine simultaneously reduced myocytes apoptosis (Fig. 2e) and infarct size (Supplementary Figure 2g), but increased the value of LVEF (Supplementary Figure 2h).

### Histamine inhibits hypoxia-induced cardiomyocytes autophagy and apoptosis

**Fig. 3:**
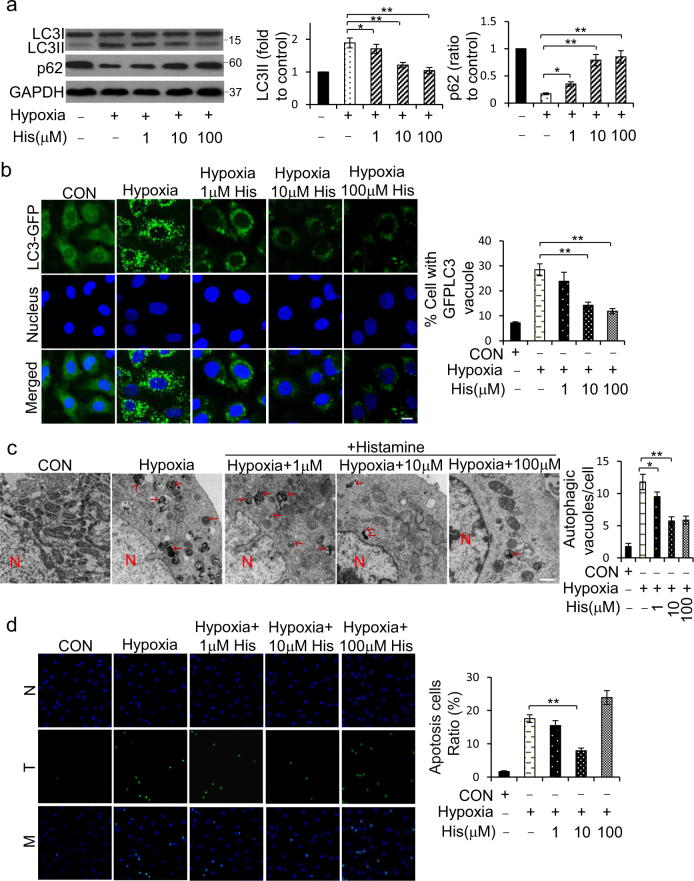
Histamine inhibits hypoxia-induced autophagy activation and apoptosis. **a** H9c2 cells were treated with histamine at the indicated dose under hypoxia for 12 h. LC3 and p62 were detected by western blotting. **b** H9c2 cells were transfected with plasmids encoding GFP-LC3 for 8 h and then treated with histamine under hypoxia at the indicated dose for 12 h. Confocal microscopy was used to visualize the appearance of autophagosomes. The ratios of autophagic cells were calculated. Scale bar = 10 μm. **c** H9c2 cells were treated as **a**. The numbers of autophagic cells were analyzed by electron microscopy (red arrows, autophagic vacuoles). Scale bar = 500 nm. **d** H9c2 cells were treated as **a**. TUNEL assay was utilized to detect apoptosis. Quantitative analysis of apoptosis is shown at the right panel. *N*, nucleus; T, TUNEL-positive cells; M, merged. Results are representative of three independent experiments. *P < 0.05; **P < 0.01

Given that lack of oxygen supply to myocardium is the salient feature of the pathophysiology of MI, we proposed to explore the role of histamine in regulating cardiomyocytes autophagy under hypoxia. The formation of LC3II and the degradation of p62 activated by hypoxia were repressed by histamine in a dose-dependent manner (Fig. 3a). The accumulation of GFP-LC3 puncta is an effective way to detect autophagosomes. Hypoxia increased GFP-LC3 puncta structures, which was significantly reduced by histamine treatment (Fig. 3b). Furthermore, EM analysis showed that histamine treatment decreased autophagic vesicles induced by hypoxia (Fig. 3c). TUNEL analysis revealed that appropriate concentration of histamine (10^−6^ ~ 10^−5^ M) could inhibit apoptosis, whereas high concentration of histamine (10^−4^ M) caused H9c2 cells to be more susceptible to apoptosis under hypoxia (Fig. 3d).

### Histamine inhibits cardiomyocytes autophagy through histamine receptor 1

**Fig. 4:**
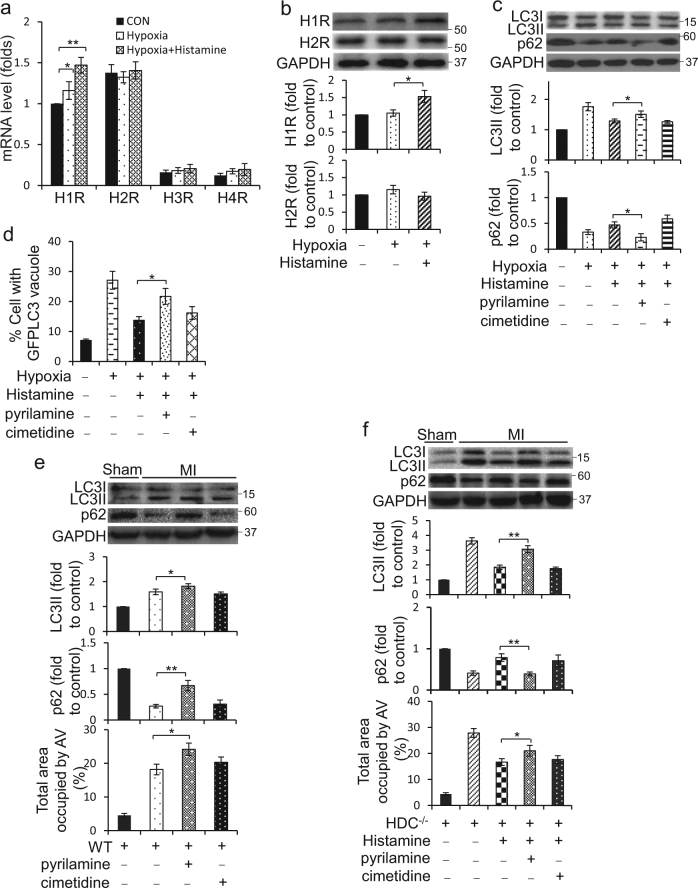
The influence of histamine on autophagy is dependent on H1R. **a** H9c2 cells were treated with 10 μM histamine under hypoxia condition for 12 h. The mRNA expression of histamine receptors was analyzed by qRT-PCR. The results were normalized to that of GAPDH. **b** H9c2 cells were treated with as **a.** The expression levels of H1R and H2R were confirmed by immunoblot. Representative photos and the densitometric analysis are shown. **c** Cells were treated with 10 μM Histamine along with 1 μM H1R antagonist (pyrilamine) or 50 μM H2R antagonist (cimetidine) for 12 h under hypoxia condition. The autophagy-related proteins, LC3 and p62, were analyzed by immunoblot and the densitometric analysis of the bands is shown. **d** H9c2 cells were transfected with plasmids encoding GFP-LC3 for 8 h and then treated as **c**. The percentage of cells with GFP-LC3 puncta was quantified. **e** From 3 days before AMI surgery, pyrilamine (10 mg/kg/d) or cimetidine (10 mg/kg/d) were administered intraperitoneally daily until euthanasia in WT mice. The levels of LC3 and p62 were analyzed by immunoblot at 3d after surgery. Representative photos and the densitometric analysis of the bands are shown as the upper panels. Quantitative analysis of autophagic cells analyzed by electron microscopy is shown at the below panel. *n* = 5 for the sham group and *n* = 8 for each of MI groups. **f** From 3 days before AMI surgery, histamine (4 mg/kg/d) with pyrilamine (10 mg/kg/d) or cimetidine (10 mg/kg/d) were administered intraperitoneally daily until euthanasia in HDC^−/−^ mice. LC3 and p62 were analyzed by immunoblot at 3d after surgery. Representative photos and the densitometric analysis of the bands are shown as the upper panels. Quantitative analysis of autophagic cells analyzed by electron microscopy is shown at the below panel. *n* = 5 for the sham group and *n* = 8 for each of MI groups. *P < 0.05; **P < 0.01

There are four known histamine receptors, H1R, H2R, H3R, and H4R, widely distributed in mammalian tissues. To figure out the very receptor participating in the regulation of histamine on autophagy, we determined the levels change of these four kinds of histamine receptors upon hypoxia or histamine treatment. The result demonstrated higher levels of H1R and H2R mRNA expressed in cardiomyocytes than H3R and H4R (Fig. 4a). However, only H1R expression significantly increased in response to histamine treatment under hypoxia (Fig. 4a, b). Pyrilamine and cimetidine are the selective antagonists for H1R and H2R, respectively. The results showed that pyrilamine significantly blocked the repression of histamine on the formation of LC3II and the degradation of p62 induced by hypoxia (Fig. 4c). Similarly, the effect of histamine on the accumulation of GFP-LC3 puncta was abolished by pyrilamine (Fig. 4d). Next, the effect of histamine and HR-antagonists on cardiomyocytes autophagy was validated in vivo. The administration of pyrilamine augmented the formation of LC3II, the degradation of p62 and the appearance of autophagic vesicles in the hearts of WT mice after MI (Fig. 4e). Likewise, the inhibition of compensatory histamine in HDC^−/−^ mice on the formation of LC3II, the degradation of p62 and the occurrence of autophagic vesicles was ruined by pyrilamine (Fig. 4f). These data indicate that the effect of histamine on cardiomyocyte autophagy was mainly H1R-dependent.

### Autophagy inhibition caused by histamine-mediated miR-206/216b elevation

**Fig. 5:**
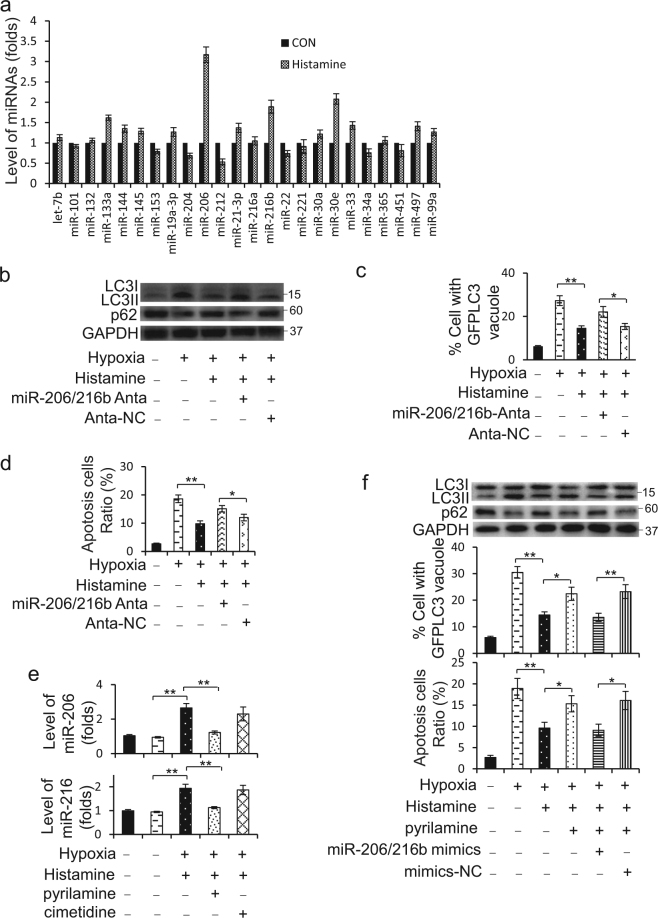
Histamine increases miR-206 and miR-216b to repress hypoxia-induced autophagy. **a** H9c2 cells were treated with 10 μM histamine. After 12 h, cells were harvested to determine the level change of miRNAs using RT-PCR. **b** H9c2 cells were transfected with miR-206 and miR-216b antagomirs together (miR-206/216b Anta) or negative controls (Anta-NC) for 8 h and then treated with 10 μM histamine under hypoxia for 12 h. LC3 and p62 were analyzed by western blotting at 3d after surgery. Representative photos are shown. **c** H9c2 cells were transfected with GFP-LC3 accompanied by miR-206 and miR-216b antagomirs (miR-206/216b Anta) or negative controls (Anta-NC). After 8 h, cells were treated with 10 μM histamine under hypoxia for 12 h. The percentage of cells with GFP-LC3 puncta was quantified. **d** H9c2 cells were transfected as **b**. Quantitative analysis of apoptosis detected by TUNEL assay is shown. **e** Cells were treated with 10 μM Histamine with 1 μM H1R antagonist (pyrilamine) or 50 μM H2R antagonist (cimetidine) for 12 h. The levels of miR-206 and miR-216b were determined by RT-PCR. **f** H9c2 cells were treated by 10 μM histamine alone or with 1 μM pyrilamine under hypoxia. After 12 h, miR-206 and miR-216b mimics mixture (miR-206/216b mimics) were transfected into cells for another 12 h. LC3 and p62 were analyzed by western blotting. Representative photos of LC3 and p62 immunoblot are shown at the above panels. Quantitative analysis of apoptosis detected by TUNEL assay is shown at the below panels. For detecting autophagy flux assay, GFP-LC3 plasmids were transfected into cells for 8 h prior to other treatment. The percentage of cells with GFP-LC3 puncta was quantified as the middle panel. *P < 0.05; **P < 0.01

We proposed to investigate whether miRNAs participate in the regulation of histamine on autophagy. First, we summarized miRNAs that have been implicated in regulating autophagy in heart (Supplementary Table 1) and detected their levels change in H9c2 cells upon histamine treatment. The results revealed the levels of miR-206, miR-30e, miR-216b, miR-133a, and miR-212 exhibited the top five significant changes upon histamine treatment, among which miR-206, miR-30e, miR-216b, and miR-133a increased, whereas miR-212 decreased (Fig. 5a; Supplementary Figure 3a). However, none of them alone significantly affected the suppression of histamine on the formation of LC3II (Supplementary Figure 3b). But knockdown of miR-206 and miR-216b (miR-206/216b) simultaneously remarkablely abolished the suppression of histamine on the formation of LC3II, the degradation of p62 (Fig. 5b; Supplementary Figure 3c), the punctate accumulations of GFP-LC3 (Fig. 5c) and apoptosis (Fig. 5d) under hypoxia. In addition, the absolute quantification of miR-206 and miR-216b in cardiomyocytes confirmed their increases upon histamine treatment (Supplementary Figure 3d). Pyrilamine reduced the levels of miR-206 and miR-216b upregulated by histamine (Fig. 5e).

Next, we wondered whether overexpression of miR-206/216b could reproduce the effect of histamine on autophagy when it is blocked. MiR-206 and miR-216b mimics were transfected into H9c2 cells to increase their levels up to 10^−5^ M histamine induced (Supplementary Figure 3e). Pyrilamine blocked the inhibition of histamine on the formation of LC3II, the degradation of p62 and the punctate accumulations of GFP-LC3 induced by hypoxia, which was recreated by miR-206/216b mimics co-transfection (Fig. 5f; Supplementary Figure 3f). Likewise, the co-transfection recapitulated the improvement of histamine on apoptosis caused by hypoxia (Fig. 5f). These data demonstrate that both miR-206 and miR-216b were the essential mediators of histamine functioning on myocardial autophagy.

### MiR-206/216b targets to Atg13

**Fig. 6:**
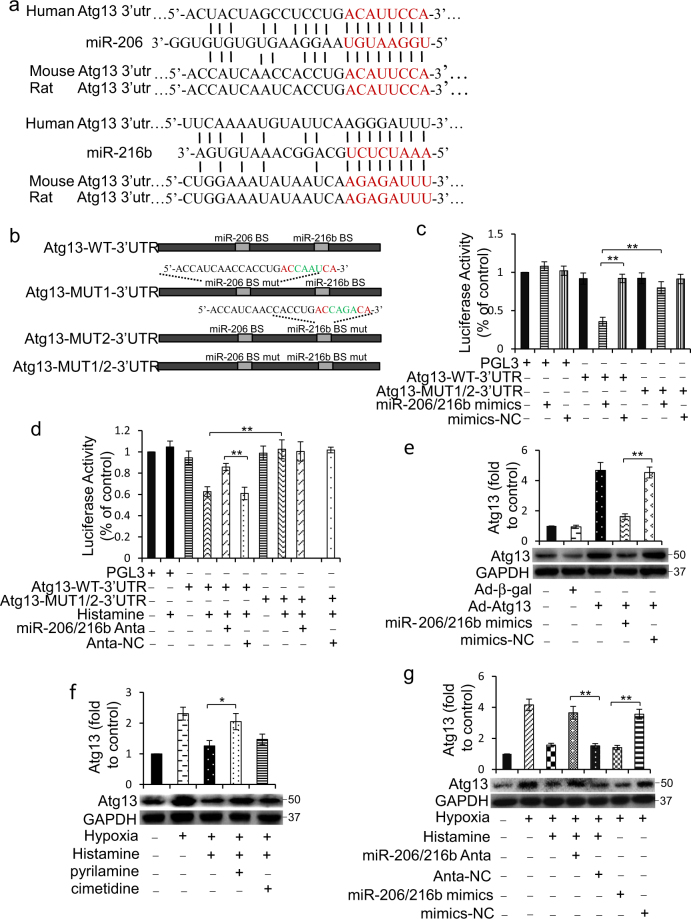

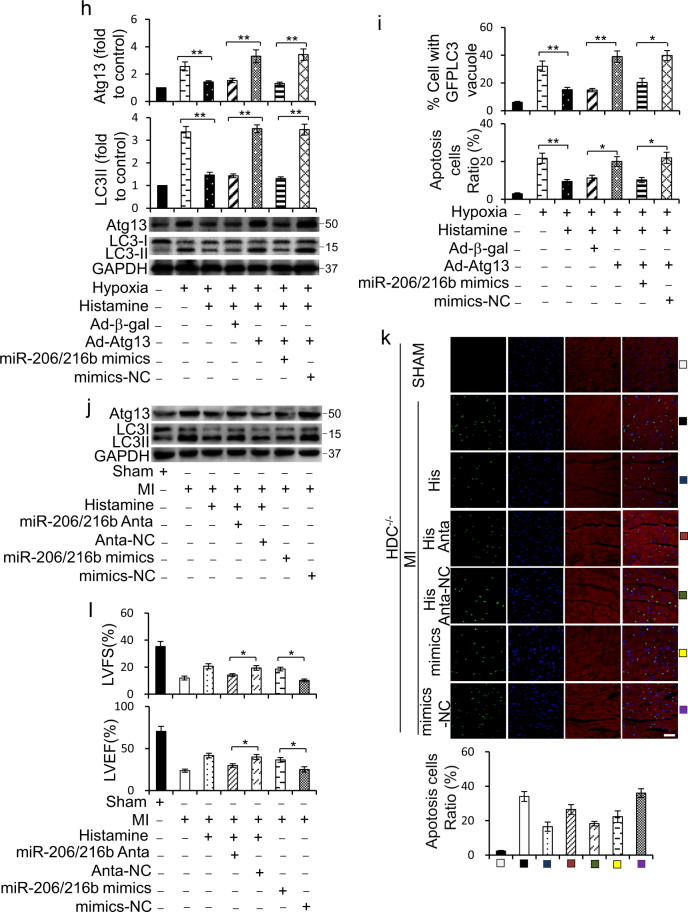
MiR-206 and miR-216b jointly target to Atg13. **a** miR-206 and miR-216b harbored conserved targeting sites in Atg13 3′-UTR. **b** Luciferase constructs of wildtype Atg13 3′-UTR (Atg13-WT-3′-UTR), mutated Atg13 3′-UTR in the miR-206-binding site (Atg13-MUT1-3′-UTR), mutated Atg13 3′-UTR in the miR-216b-binding site (Atg13-MUT2-3′-UTR), and mutated Atg13 3′-UTR in the miR-206/216b binding sites (Atg13-MUT1/2–3′UTR) are shown. **c** HEK293 cells were infected with miR-206/216b mimics or mimics negative control (mimics-NC) along with the luciferase constructs of Atg13-WT-3′-UTR, Atg13-MUT1/2–3′UTR, or the empty vector pGL3. Luciferase activity was measured after 24 h. **d** H9c2 cells were infected with luciferase constructs of Atg13-WT-3′-UTR, Atg13-MUT1/2–3′UTR, or the empty vector pGL3 along with miR-206/216b antagomirs (miR-206/216b Anta) or antagomir negative control (Anta-NC) for 8 h. Cells were then treated with 10 μM histamine for 12 h. Luciferase activity was measured. **e** H9c2 cells were infected with adenoviral constructs expressing Atg13 (Ad-Atg13) or β-gal (Ad-β-gal) along with miR-206/216b mimics or negative control (mimics-NC). The level of Atg13 was analyzed by immunoblot. **f** H9c2 cells were treated with 10 μM histamine with 1 μM pyrilamine or 50 μM cimetidine under hypoxia for 12 h. The level of Atg13 was analyzed by immunoblot. **g** MiR-206/216b antagomirs (miR-206/216b Anta) or their negative controls (Anta-NC) were transfected into H9c2 cells for 8 h. Then cells were treated with 10 μM histamine under hypoxia for 12 h. For overexpression of miR-206/216b, H9c2 cells were transfected with miR-206/216b mimics or mimics-NC for 8 h. The level of Atg13 was analyzed by immunoblot. **h** H9c2 cells were infected with adenoviral constructs expressing Atg13 (Ad-Atg13) or β-gal (Ad-β-gal) along with miR-206/216b mimics or mimics-NC for 8 h. Then cells were treated with 10 μM histamine under hypoxia for 12 h. Representative photos of LC3 and p62 immunoblot and the densitometric analysis of the bands are shown. **i** Cells were treated as **h**. Quantitative analysis of apoptosis detected by TUNEL assay is shown. For detecting autophagy flux assay, GFP-LC3 plasmids were transfected into cells for 8 h prior to other treatment. The percentage of cells with GFP-LC3 puncta was quantified as the middle panel. **j** HDC^−/−^ mice were intravenously injected for 3 consecutive days before MI with miR-206/216b mimics, their antagomirs (miR-206/216b Anta), or their negative controls as described in methods along with 4 mg/kg/d histamine until euthanasia. Atg13 and LC3II levels were detected by immublot. **k** HDC^−/−^ mice were treated as **j**. Myocardial cell apoptosis was analyzed by TUNEL assay. TUNEL-positive nuclei (apoptotic cells) are green. Nuclei stained by DAPI show blue. Cardiomyocytes were labeled with a-actinin. scale bar, 50 μm. *n* = 3 for the sham group and *n* = 3 for each of MI groups. **l** HDC^−/−^ mice were treated as **j**. Cardiac function 1 week after MI surgery was analyzed by echocardiographic analysis. LVEF, left ventricular eject fraction. LVFS: left ventricular fraction shortening. *n* = 4 for the sham group and *n* = 4 for each of MI groups. *P < 0.05; **P < 0.01

We consequently used TargetScan to analyze the potential targets of miR-206 and miR-216b in autophagy. Interestingly, we noticed that both miR-206 and miR-216b possessed conserved binding sites on 3′-UTR of Atg13 (Fig. 6a). To confirm whether Atg13 is really a direct target of miR-206 or miR-216b, we created the luciferase constructs with a wildtype Atg13 3′-UTR (Atg13-WT-3′-UTR), with a miR-206-binding site-mutated Atg13 3′-UTR (Atg13-MUT1-3′-UTR), with a miR-216b-binding site-mutated Atg13 3′-UTR (Atg13-MUT2-3′-UTR) or with two binding sites-mutated Atg13 3′-UTR (Atg13-MUT1/2–3′UTR) (Fig. 6b). Luciferase assay revealed that both miR-206 and miR-216b could reduce the luciferase activity of Atg13-WT-3′-UTR (Supplementary Figure 4a and 4b). Even so, miR-206/216b co-transfection caused a more extraordinary decrease in the luciferase activity of Atg13-WT-3′-UTR (Fig. 6c). Histamine repressed the activity of luciferase constructs of Atg13-WT-3′-UTR under hypoxia, and this was attenuated by miR-206 and miR-216b antagomir co-transfection (Fig. 6d). However, these effects could not be observed in luciferase constructs of Atg13-MUT1/2–3′UTR (Fig. 6d). Next, we infected cells with adenovirus harboring Atg13 with wildtype 3′-UTR along with miR-206, miR-216b, miR-206/216b mixture, or negative control. Unexpectedly, miR-206 or miR-216b alone has no significant effect on the level of Atg13 protein (Supplementary Figure 4c and 4d), whereas miR-206/216b mixture remarkablely subdued the translation of Atg13 (Fig. 6e). These data suggested Atg13 as a co-target gene of miR-206 and miR-216b acting in concert.

In addition, the level of Atg13 sharply decreased upon histamine treatment, which was canceled by pyrilamin (Fig. 6f). Knockdown of miR-206 and miR-216b simultaneously could raise Atg13 expression reduced by histamine under hypoxia, whereas overexpression of miR-206/216b cut down Atg13 level (Fig. 6g). Overexpression of Atg13 could abolish the suppression of histamine on the formation of LC3II (Fig. 6h), the formation of GFP-LC3 granule and apoptosis caused by hypoxia (Fig. 6i). However, this effect of Atg13 overexpression was countermanded by miR-206/216b mimics (Fig. 6h,i).

The role of miR-206 and miR-216b in histamine signaling was further confirmed in vivo. Deletion of miR-206 and miR-216b simultaneously could attenuate the prohibitive effect of histamine on Atg13 level, LC3II formation (Fig. 6j; Supplementary Figure 4e), and cell death (Fig. 6k), as well as abolished the protective effect of histamine on myocardial function in response to MI in HDC^−/−^ mice (Fig. 6l). Conversely, overexpression of miR-206/216b could simulate the effect of histamine on Atg13 level, LC3II formation (Fig. 6j), cell death (Fig. 6k), and myocardial function (Fig. 6l) in HDC^−/−^ MI mice.

### Atg13 interacts with FADD to promote myocardial apoptosis

**Fig. 7:**
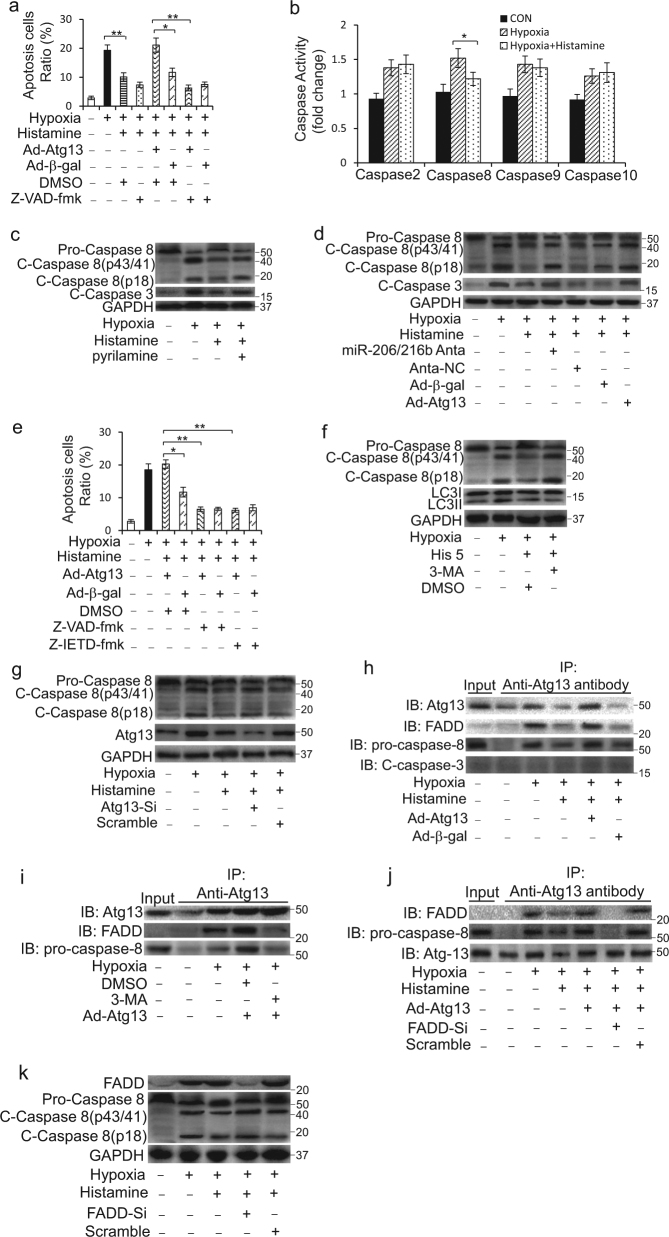
Atg13 interacts with FADD to activate caspase-8 under hypoxia. **a** H9c2 cells were infected with adenoviral constructs expressing Atg13 (Ad-Atg13) or β-gal (Ad-β-gal) and then treated with 10 μM histamine accompanied by DMSO or 20 μM Z-VAD-fmk for 24 h. Quantitative analysis of apoptosis detected by TUNEL assay is shown. **b** H9c2 cells were treated with 10 μM histamine under hypoxia for 24 h. The activities of caspases as indicated were determined by the corresponding kits. **c** H9c2 cells were treated with 10 μM histamine along with 1 μM pyrilamine under hypoxia for 24 h. The activation of caspase-8 and caspase-3 was determined by immunoblot. **d** H9c2 cells were infected with adenoviral Atg13 (Ad-Atg13) or were transfected with miR-206/216b antagomirs (miR-206/216b Anta) for 8 h prior to 10 μM histamine treatment under hypoxia for 12 h. The activation of caspase-8 and caspase-3 was determined by immunoblot analysis. **e** H9c2 cells were infected with adenoviral Atg13 (Ad-Atg13) or β-gal (Ad-β-gal) and then treated with 10 μM histamine accompanied by DMSO as control, 20 μM Z-VAD-fmk or 10 μM Z-IETD-fmk for 24 h. Quantitative analysis of apoptosis detected by TUNEL assay is shown. **f** H9c2 cells were treated with 10 μM histamine and 2 mM 3-methyladenine (3-MA) under hypoxia for 24 h. The activation of caspase-8 and the formation of LC3II were determined by immunoblot. **g** H9c2 cells were treated with 110 nM Atg13-siRNA (Atg13-Si) or its negative controls (Scramble) for 8 h prior to 10 μM histamine treatment for 24 h. The activation of caspase-8 and the level of Atg13 were determined by immunoblot. **h** H9c2 cells were infected with adenoviral Atg13 (Ad-Atg13) or β-gal (Ad-β-gal) and then treated with 10 μM histamine under hypoxia for 24 h. Immunoprecipitation was performed using an Atg13 antibody. The anti-IgG served as a control. The levels of Atg13, FADD, pro-caspase, and cleaved caspase-3 were analyzed by immunoblot. **i** H9c2 cells were infected with adenoviral Atg13 (Ad-Atg13) for 8 h prior to 2 mM 3-methyladenine (3-MA) treatment under hypoxia for 24 h. Immunoprecipitation was performed using an Atg13 antibody. The anti-IgG served as a control. The levels of Atg13, FADD, and pro-caspase were analyzed by immunoblot.  **j** H9c2 cells were infected with adenoviral Atg13 (Ad-Atg13) accompanied by 90 nM FADD siRNA (FADD-Si) or its negative controls. Then cells were treated with 10 μM histamine under hypoxia for 24 h. Immunoprecipitation was performed using an Atg13 antibody. The anti-IgG served as a control. The levels of Atg13, FADD and pro-caspase were analyzed by immunoblot. **k** H9c2 cells were transfected with 90 nM FADD-Si or its negative controls. Then cells were treated with 10 μM histamine under hypoxia for 24 h. The activation of caspase-8 and the level of FADD were determined by immunoblot. *P < 0.05; **P < 0.01

Given the complicated relationships between autophagy and apoptosis, we aimed to explore the mechanisms involving in the regulation of histamine on these two processes. Apoptosis is defined by the activation of a family of caspases^6^. Enforced expression of Atg13 abolished the suppression of histamine on cell death caused by hypoxia, but the cell death was efficiently reduced by co-treatment with Z-VAD-fmk, a pan-caspase inhibitor (Fig. 7a; Supplementary Figure 5a). These data suggested that caspases activation was involved in the histamine-Atg13 signal regulated cell death. Caspase-2, Caspase-8, Caspase-9, and Caspase-10 are initiators and caspase-3 is the most important executor of apoptosis. Histamine attenuated the activity of caspase-8 in the context of hypoxia (Fig. 7b). Immunoblots data showed that histamine significantly reduced the cleavage of caspase-8 and caspase-3 caused by hypoxia, which was abolished by pyrilamine (Fig. 7c). Knockdown of miR-206/216b or overexpression of Atg13 attenuated the effect of histamine on the cleavage of caspase-8 and caspase-3 (Fig. 7d; Supplementary Figure 5b). Moreover, Z-IETD-fmk, a caspase-8-specific inhibitor, could lessen Atg13 overexpression-induced cell death to an approximate level as Z-VAD-fmk did (Fig. 7e).

Histamine inhibited the cleavage of caspase-8 accompanied with its repression on the formation of LC3II (Fig. 7f; Supplementary Figure 5c). But after autophagy activity was inhibited by 3-MA, histamine could not subdue the cleavage of caspase-8 caused by hypoxia anymore (Fig. 7f; Supplementary Figure 5c). Similarly, deletion of Atg13 by Atg13 siRNA attenuated the effect of histamine on caspase-8 activation (Fig. 7g; Supplementary Figure 5d).

As Atg12-Atg5 complex interacts with the death adapter protein FADD to activate caspase-8^38^, so we explored whether FADD participated in the cleavage of caspase-8 regulated by histamine. Histamine treatment had no effect on the expression of FADD (Supplementary Figure 5e). Co-immunoprecipitation analysis demonstrated that Atg13 had an interaction with FADD and/or pro-caspase-8 under hypoxia (Fig. 7h and Supplementary Figure 5f). But the interaction was interrupted by 3-MA treatment (Fig. 7i; Supplementary Figure 5g). In addition, after FADD was deleted by FADD siRNA, Atg13 had no interaction with pro-caspase-8 (Fig. 7j; Supplementary Figure 5h) and histamine could not restrain the activation of caspase-8 and caspase-3 caused by hypoxia anymore (Fig. 7k; Supplementary Figure 5i). Moreover, FADD deletion canceled the repression of histamine on apoptosis and abolished apoptosis induced by overexpression of Atg13 under hypoxia (Supplementary Figure 5j). These results indicate that Atg13 interacts with FADD to activate caspase-8 through which histamine regulates apoptosis under hypoxia.

Beclin 1 (ATG6) is required for the formation of autophagasome in autophagy^39^. To further confirm whether the protective effect of histamine on cardiac injury is dependent on its regulatory role in apoptosis and autophagy, we tested the effects of Z-IETD-fmk, adenoviral Beclin 1 siRNA and Atg13 siRNA on cardiac dysfunction and cell death in HDC^−/−^ mice. Atg13, Beclin 1, LC3II, and p62 levels were monitored (Supplementary Figure 5k and 5l). The administration of Z-IETD-fmk, adenoviral Beclin 1 siRNA, or Atg13 siRNA could attenuate cell death observed in HDC^−/−^ mice (Supplementary Figure 5m), as well as ameliorated myocardial function in response to MI in HDC^−/−^ mice (Supplementary Figure 5n).

## Discussion

We demonstrate here that histamine plays critical roles in regulating cardiomyocyte autophagy and apoptosis during AMI. Histamine levels increase in the serum of patients and murine model with AMI accompanied by the increment of HDC-expressing CD11b^+^ myeloid cells. HDC-expressing myeloid cells derived-histamine seems to inhibit hypoxia-induced cardiomyocytes autophagy and apoptosis through H1R signal. Furthermore, miR-206/216b-Atg13 axis has been identified as the intracellular signal of histamine exerting autophagic effect under hypoxia.

Previous study reported that the unique enzyme HDC for histamine production is highly expressed in CD11b^+^Gr-1^+^ immature myeloid cells within the bone marrow and histamine deficiency promotes inflammation-associated colonic tumorigenesis in HDC^−/−^ mice^36^. Using HDC-EGFP transgenic mice, HDC-expressing CD11b^+^ myeloid cells have been demonstrated as the persistent source of endogenous histamine in response to inflammatory stimuli, such as colitis and AMI^30, 36^. In the current study, the results further confirmed the increase of HDC mRNA in circulating CD11b^+^ myeloid cells of AMI mice and patients.

That histamine elevated in serum from ischemic heart disease patients is wildly recognized^29, 40^. But the roles of histamine or histamine receptors in ischemic heart disease have not been fully clarified. In contrast to dogma that histamine was thought to be a proinflammatory molecule in acute coronary syndrome^41^, our recent work demonstrated that histamine deficiency aggregated cardiac dysfunction associated with increased cardiomyocyte apoptosis in the early stages of AMI and myocardial fibrosis during the repair of AMI^30, 42^. Deng et al. ^30^ reported that histamine inhibits cardiomyocyte apoptosis through the activation of H1R- and H2R-related signals and histamine deletion leads to abnormal infiltration and differentiation of macrophages. Consistent with the protective effect of histamine against cardiac ischemia, histamine has been reported to alleviate neuronal damage and infarct volume^32, 33, 43, 44^. However, further studies are needed to delineate the precise mechanisms involved in the protection of histamine in ischemic diseases.

Interestingly, we observed that histamine deficiency in HDC^−/−^ mice augmented cardiomyocytes apoptosis accompanied by enhanced autophagy activation post AMI. Unlike H1R and H2R, which are both involved in myocardial apoptosis, our results demonstrated that the autophagic effect of histamine was conveyed mainly through H1R. The potential role of H1R in autophagic process had also been noted by other groups. H1R antagonists such as diphenhydramine, pyrilamine, and astemizole induced vacuolation in vascular smooth muscle cell^45^ and astrocytes^46^, which were accompanied by the increase of LC3II. However, Jakhar et al.^47^ reported that histamine combined with H1R antagonist Astemizole triggered endoplasmic reticulum stress-induced apoptotic cell death along with Beclin 1-independent autophagy induction in breast cancer cells, which seems to be inconsistent with our study. This may due to in different cell types and under different pathological conditions.

A lot of studies have reported the dual nature of autophagy in ischemic cardiovascular diseases. Some researches revealed that the inhibition of the early steps of autophagosome formation will reduce apoptosis, whereas the inhibition of the late steps of autophagy shows an opposite effect^48, 49^. Autophagosome formation requires a series of autophagy-related proteins^50^, among which Atg1/ULK complex is one of the most upstream factors of autophagosome formation. Atg13 is an essential member of Atg1/ULK complex. Autophagy and apoptosis share many common upstream pathways and can regulate each other^51^. In our work, it was observed that histamine deficiency caused the increase of Atg13 expression, which facilitated to activate caspase-8 to aggravate apoptosis in the peri-infarct area caused by AMI.

Besides necrosis, apoptosis also significantly contributes to cardiomyocytes death in AMI and occurs predominantly in the peri-infarcted region. Patients who developed symptomatic heart failure shortly after AMI were associated with significantly increased apoptotic rates^4^. Necrosis has been proposed as a caspase-independent programmed cell death. In our work, the administration of the caspase-8-specific inhibitor Z-IETD-fmk could abolish the exacerbation of cardiac dysfunction and cell death observed in HDC^−/−^ mice post AMI, hinting that the protective function of histamine is mainly to inhibit cellular apoptosis but not necrosis caused by AMI. In addition, our in vitro experiments revealed that modest concentration of histamine could inhibit cardiomyocytes apoptosis, whereas overdose of histamine aggravated apoptosis caused by hypoxia. This may be due to that overdose of histamine repressed autophagy activity to an extent harmful for cell survival under hypoxia.

Growing evidence has shown that miRNA can regulate autophagy^52^. miRNA could function on its own or work together with other miRNAs^53, 54^. Our data showed histamine increased miR-206 and miR-216b simultaneously. Atg13 was their co-target. One of them alone could not recreate histamine autophagic effect or influence the protein level of Atg13, implying miR-206 and miR-216b act in concert to repress the translation of Atg13. Further investigation will focus on how histamine increases the levels of miR-206/216b and how these two miRNAs act concordantly to regulate Atg13 expression.

Mounting evidence has suggested that autophagy is implicated in the induction of caspase-dependent cell death^55, 56^. It has recently been suggested that caspase-8 is recruited to autophagosomal membrane through FADD and that autophagic machinery is required for activation of caspase-8^57^. In the present study, it was observed that Atg13 interacted with FADD to activate caspase-8. But when autophagy was inhibited by 3-MA, Atg13 could not interact with FADD anymore, implying the interaction of Atg13 and FADD requiring other autophagic factors. Further studies investigating the factors facilitating the interaction of Atg13 and FADD will be required.

Acute coronary syndromes might cause cardiac hypoxia and hemodynamic abnormalities. Strategies that could attenuate the bad consequence of hypoxia and hemodynamic abnormalities have been considered potentially beneficial for the treatment of AMI. Our present work only prompted that the modest dose of histamine implemented its protective effect on MI at least partially through suppressing autophagy and apoptosis caused by hypoxia, but cannot exclude the likelihood that the benefit effect of histamine is also partially due to the protection against hemodynamic stress.

**Fig. 8:**
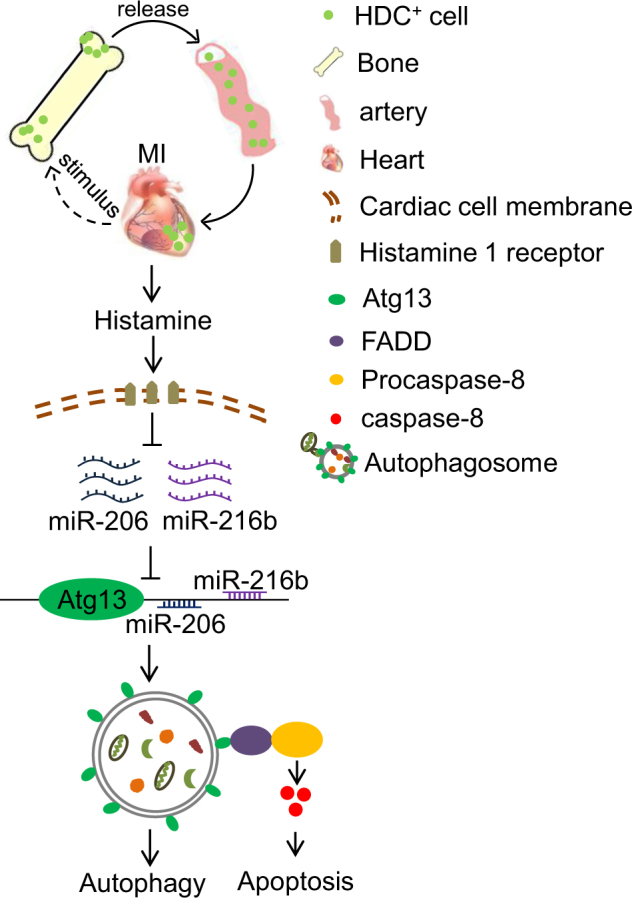
Schematic model of histamine-mediated signal in regulating cardiomyocyte autophagy and apoptosis in the context of acute myocardial infarction

In conclusion, our data clearly indicate that histamine plays a prohibitive role in MI-caused myocardial apoptosis and autophagy activation. Histamine increases miR-206 and miR-216b expression, which work combined to downregulate Atg13, resulting in the reduction of autophagy. The interaction of Atg13 with FADD to activate caspase-8 is a crosstalk of autophagy with apoptosis, leading to autophagy-dependent apoptosis repressed by histamine (Fig. 8). Our results highlight important roles of histamine in the protection against ischemic myocardial injury and suggest novel therapeutic targets for the applications of histamine or histamine receptor antagonists.

## Electronic supplementary material

supplementary figures

supplementary figure legends

sumpplementary table 1
