# Supplementary figures and images for: Histamine deficiency aggravates cardiac injury through miR-206/216b-Atg13 axis-mediated autophagic-dependant apoptosis
Source: Cell Death Dis. 2018 Jun 7;9(6):694. doi: 10.1038/s41419-018-0723-6 (PMC5992227; doi:10.1038/s41419-018-0723-6)

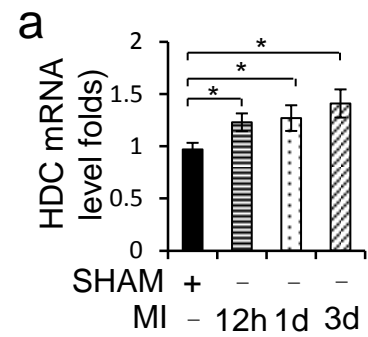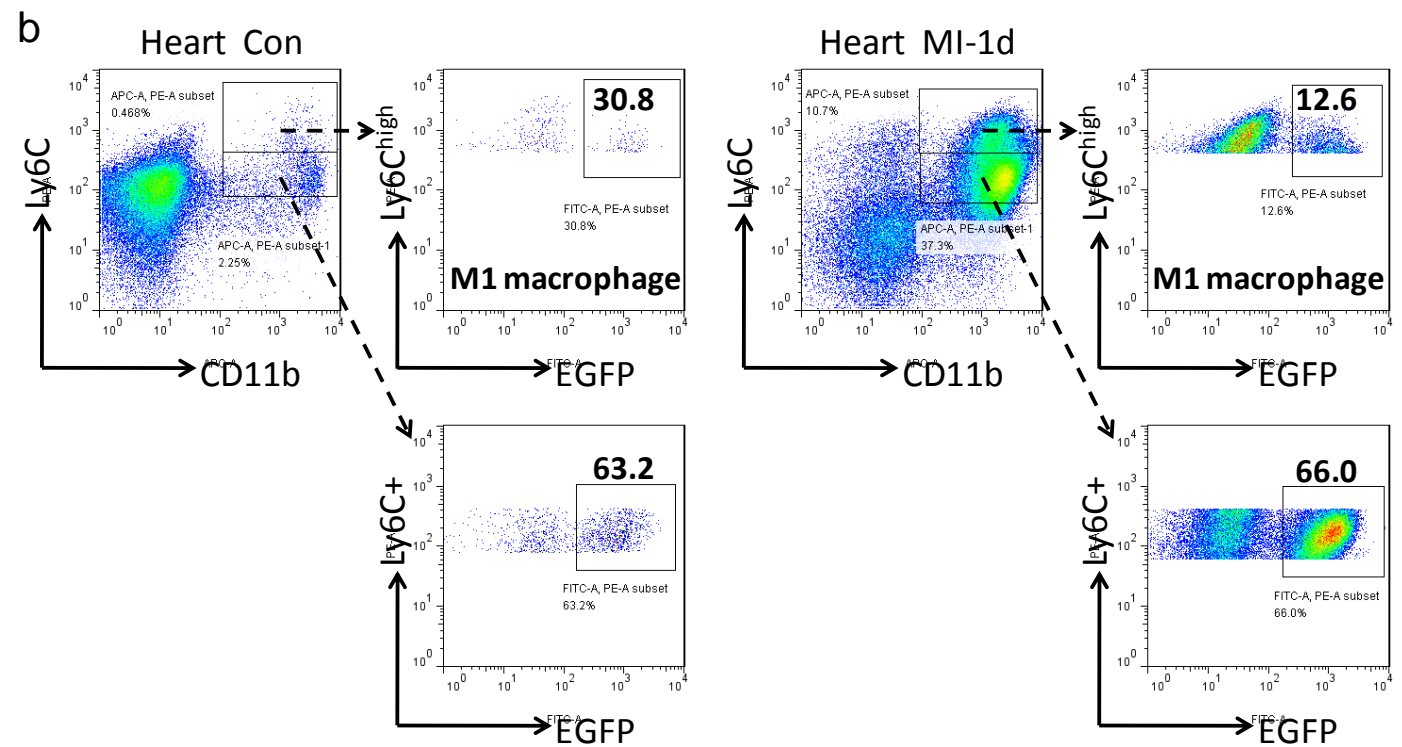

Supplementary Figure 2

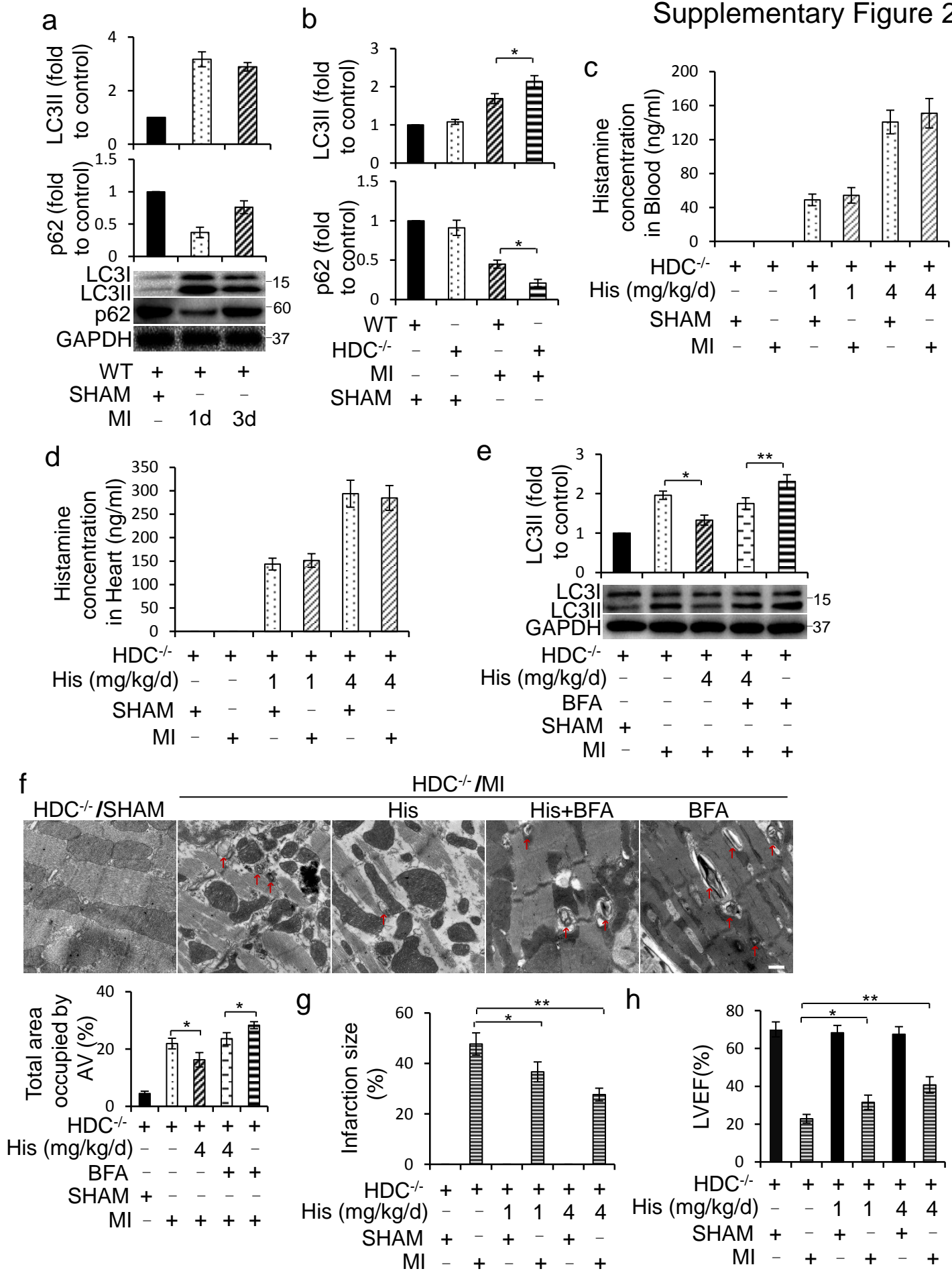

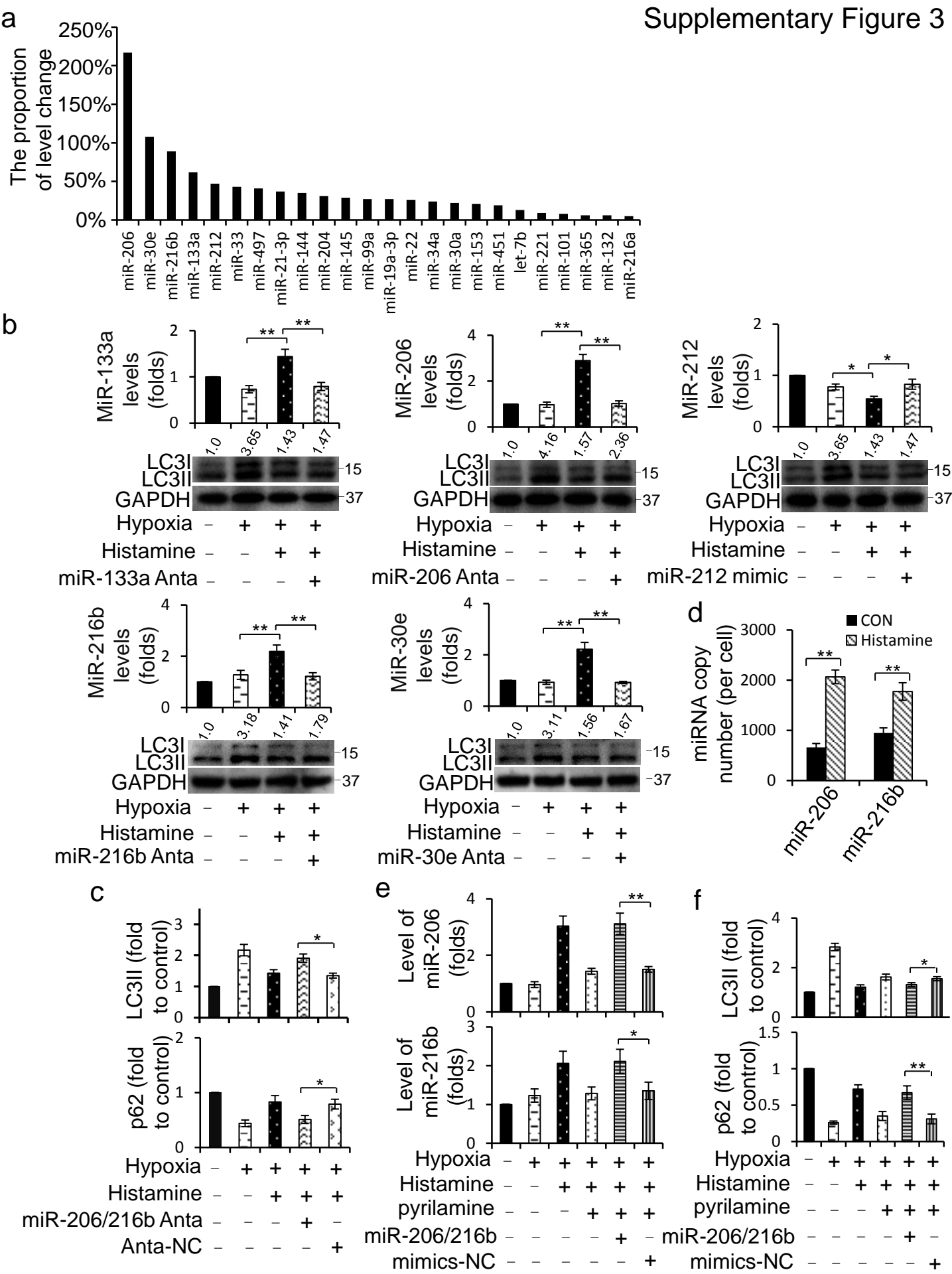

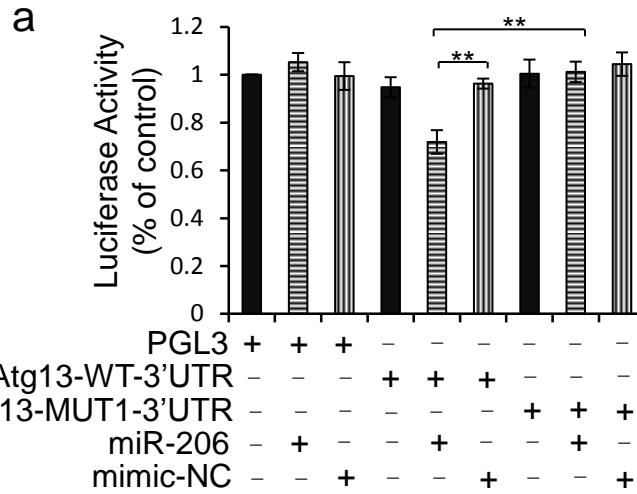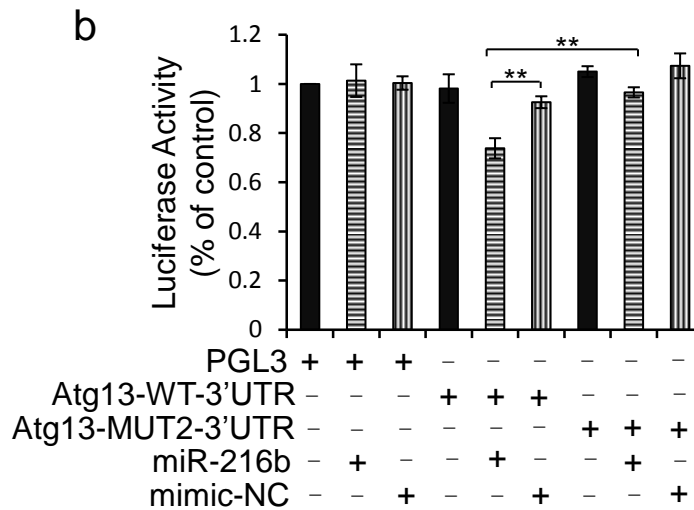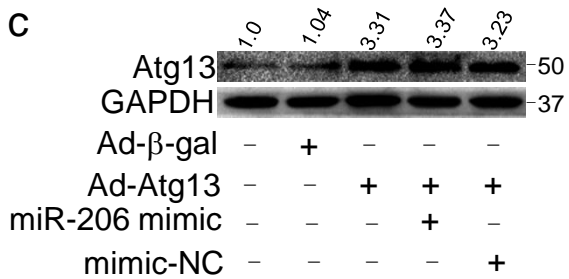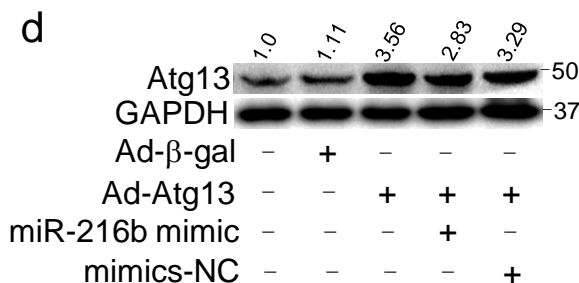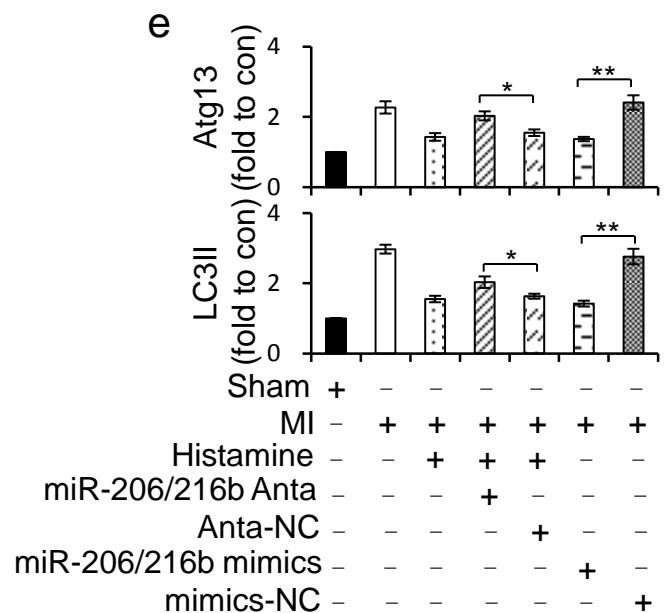

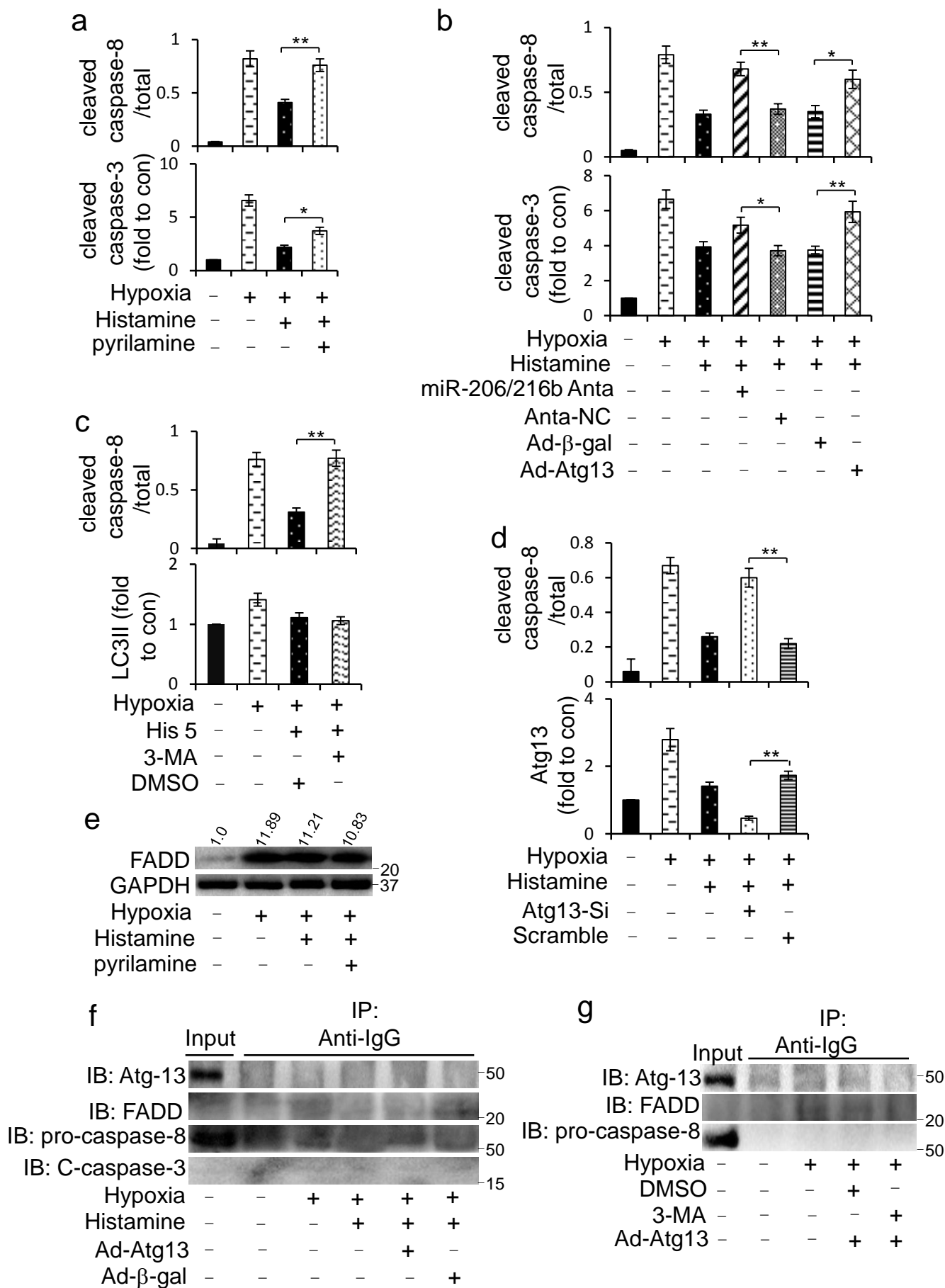

h

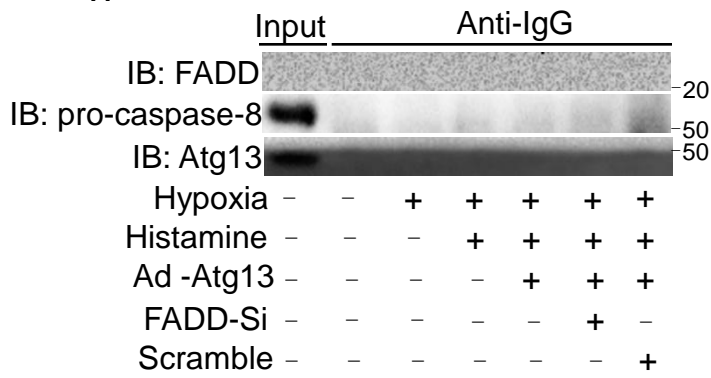

j

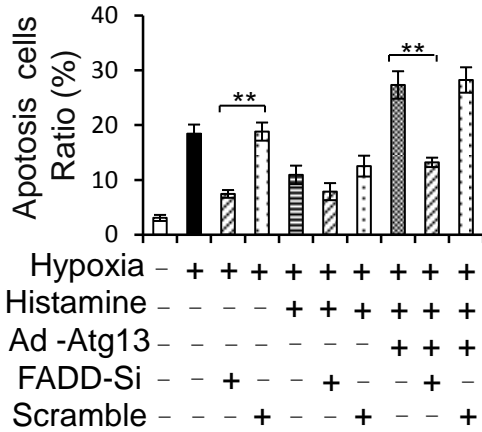

l

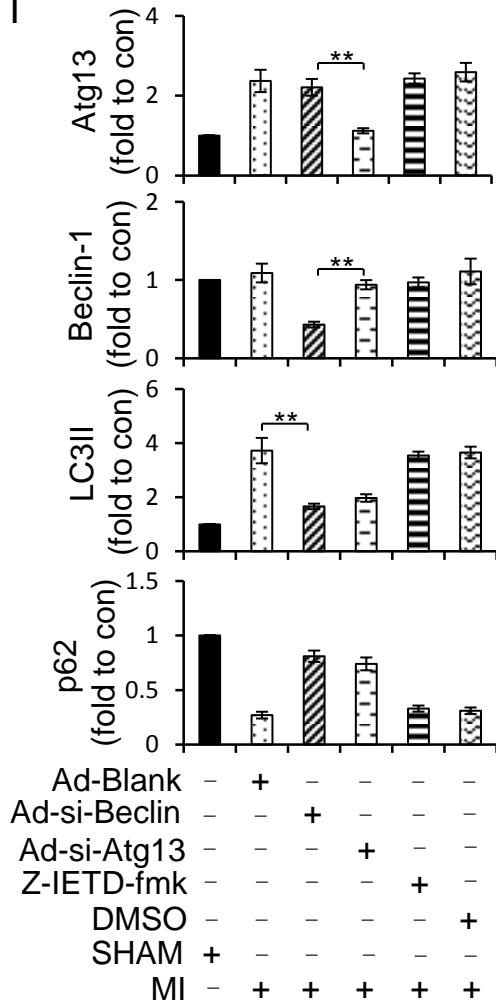

i

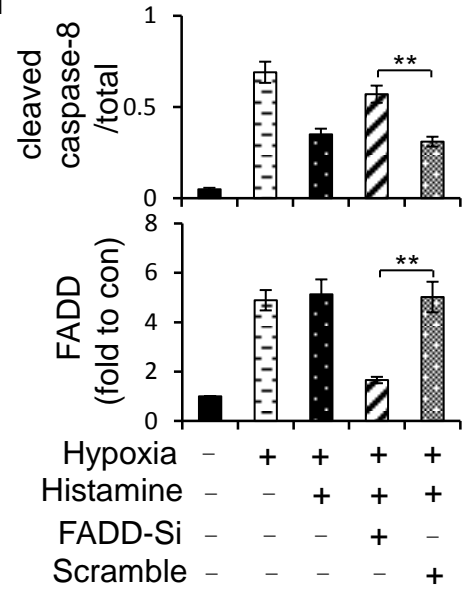

k

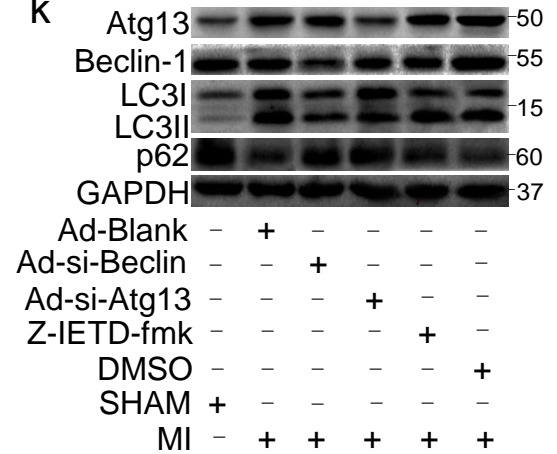

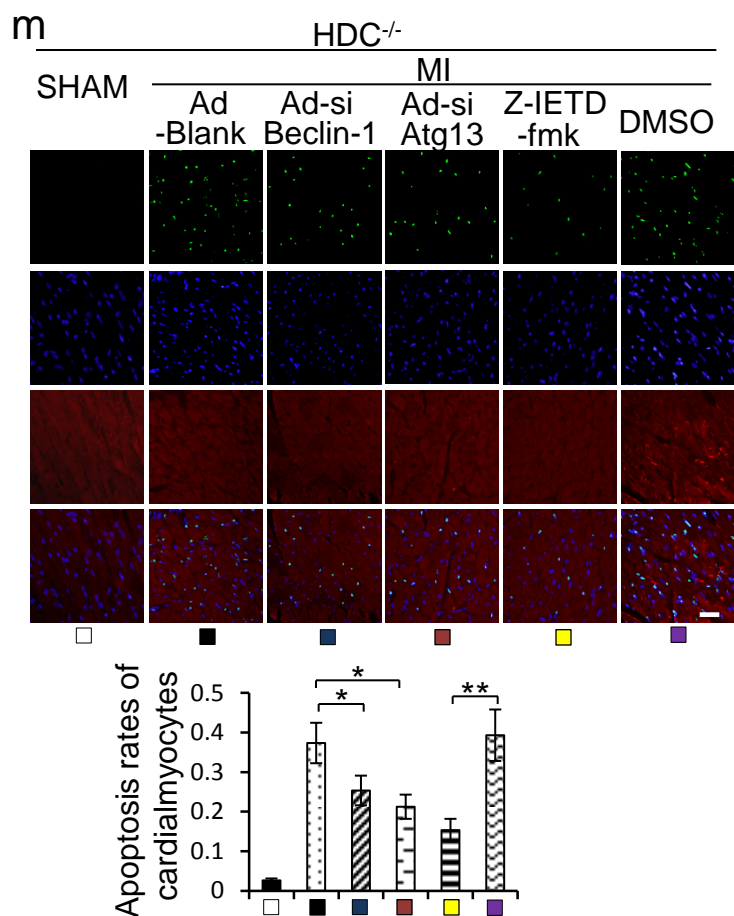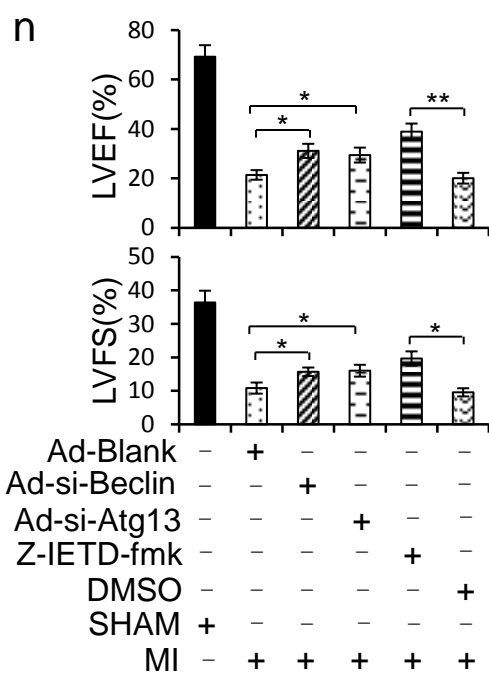

Supplement: Supplementary file 1 — supplementary figures [file 41419_2018_723_MOESM1_ESM.pdf]
