# Supplementary material for: Histamine deficiency aggravates cardiac injury through miR-206/216b-Atg13 axis-mediated autophagic-dependant apoptosis
Source: Cell Death Dis. 2018 Jun 7;9(6):694. doi: 10.1038/s41419-018-0723-6 (PMC5992227; doi:10.1038/s41419-018-0723-6)
Supplement: Supplementary file 2 — supplementary figure legends [file 41419_2018_723_MOESM2_ESM.pdf]

Supplementary Figure Legends

Supplementary Figure 1 Histamine levels increased and histamine deficiency aggravated myocardial apoptosis after myocardial infarction. (a) HDC mRNA level in the blood mononuclear cells of AMI mice model at indicated day after AMI surgery was analyzed by qRT-PCR (n=5 for the sham group and n=8 for each of the MI groups). (b) The percentage of $EGFP^{+}$ cells in $CD11b^{+}Ly6C^{high}$ M1-type macrophages in the hearts of WT mice post MI was determined by FACS with myeloid cell surface markers anti-CD11b and anti-Ly6C (n=5 for the sham group and n=8 for each of the MI groups). Data are presented as mean±s.e.m. and analysis was performed with one-way ANOVA followed by Tukey–Kramer post hoc analysis. *P<0.05; **P<0.01.

Supplementary Figure 2 Histamine suppresses autophagy overactivation and cell death in cardiomyocytes in AMI $HDC^{-/-}$ mice model. (a) The level of LC3 in hearts was analyzed by immunoblot. Representative photos and densitometric analysis of the Western blot bands are shown. (b) Densitometric analysis of the Western blot bands of the autophagy-related proteins LC3 and p62 in the hearts at 3d after AMI surgery (n=5 for each group). (c and d) HDC deficiency mice were administered exogenous histamine intraperitoneally for 3 consecutive days at the dose of 1mg/kg or 4mg/kg before exposed to AMI surgery until euthanasia. Histamine concentration in the serum (c) and heart (d) was determined at 3 d post AMI by histamine Elisa kit (n=5 for each group). (e) HDC deficiency mice were administered 4mg/kg exogenous histamine and/or 0.3 mg/kg bafilomycin A1 intraperitoneally for 3 consecutive days before

exposed to AMI surgery until euthanasia. The level of LC3 in hearts was analyzed by immunoblot (n=3 for each group). (f) $HDC^{-/-}$ mice were treated as in (e). Representative electron micrographs (EM) of hearts from $HDC^{-/-}$ mice at 3d after AMI surgery. Bar=200nm. Quantification of autophagic vacuoles (red arrows) is shown (n=3 for each group). (g) $HDC^{-/-}$ mice were treated as in (c). Echocardiographic analysis of cardiac function at 1 week after AMI surgery. n=5 for each group. (h) $HDC^{-/-}$mice were treated as (c). Infarct size was analyzed by TTC-staining at 3d after AMI surgery. n=6 for each group. Data are presented as mean ±s.e.m. and analysis was performed with one-way ANOVA followed by Tukey–Kramer post hoc analysis. *P<0.05; **P<0.01.

Supplementary Figure 3 Histamine increases miR-206 and miR-216b to repress hypoxia induced autophagy. (a) miRNAs levels were shown as descending order of their levels change upon histamine treatment. Results are representative of three independent experiments. (b) H9c2 cells were transfected with miR-206 antagomir (miR-206 Anta), miR-30e antagomir (miR-30e Anta), miR-216b antagomir (miR-216b Anta), miR-133a antagomir (miR-133a Anta) or miR-212 mimics for 12h and then treated with 10μM histamine under hypoxia for another 12h. Cells were harvested for determining LC3II formation by immunoblot and miRNA level by RT-PCR. Results are representative of three independent experiments. (c) H9c2 cells were transfected with miR-206 and miR-216b antagomirs (miR-206/216b Anta) together or negative controls (Anta-NC) for 12h and then treated with 10μM

histamine under hypoxia for 12h. LC3 and p62 were analyzed by western blotting at 3d after surgery. The densitometric analysis of the bands are shown. (d) Cardiomyocytes isolated from 1-2 days old mice heart. Cells were treated with 10μM histamine for 12h. The copy numbers of miR-206 and miR-216b in per cardiomyocyte were detected by absolute quantification RT-PCR. Results are representative of three independent experiments. (e) H9c2 cells were transfected with miR-206/216b mimics simultaneously for 12h and then treated with 10μM histamine with 1μM pyrilamine under hypoxia for another 12h. MiR-206 and miR-216b levels were detected by RT-PCR. Results are representative of three independent experiments. (f) H9c2 cells were treated by 10μM histamine alone or with 1μM pyrilamine under hypoxia. After 12h, miR-206 and miR-216b mimics mixture (miR-206/216b mimics) was transfected into cells for another 12h. LC3 and p62 were analyzed by western blotting. The densitometric analysis of the bands are shown. Data are presented as mean±s.e.m. and analysis was performed with one-way ANOVA followed by Tukey–Kramer post hoc analysis. *$P<0.05$; **$P<0.01$.

Supplementary Figure 4 MiR-206 and miR-216b jointly target to Atg13. (a) HEK293 cells were infected with miR-206 mimic or mimic-NC for 12h, and then transfected with the luciferase constructs of Atg13-WT-3'UTR, Atg13-MUT1-3'UTR or the empty vector pGL3. Luciferase activity was measured after 24h. Results are representative of three independent experiments. (b) HEK293 cells were infected with miR-216b mimic or mimic-NC for 12h, and then transfected with the luciferase

constructs of Atg13-WT-3’UTR, Atg13-MUT2-3’UTR or the empty vector pGL3. Luciferase activity was measured after 24h. Results are representative of three independent experiments. (c) H9c2 cells were infected with adenoviral constructs expressing Atg13 (Ad-Atg13) or β-gal (Ad-β-gal) along with miR-206 mimic or mimic-NC. The level of Atg13 was analyzed by immunoblot. Results are representative of three independent experiments. (d) H9c2 cells were infected with adenoviral Atg13 (Ad-Atg13) or β-gal (Ad-β-gal) along with miR-216b mimic or mimic-NC. The level of Atg13 was analyzed by immunoblot. Results are representative of three independent experiments. (e) $HDC^{-/-}$ mice were intravenously injected for three consecutive days before MI with miR-206/216b mimics, antagomir (Anta) or their negative controls (NC) until euthanasia as described in methods along with 4mg/kg/d histamine, and then subjected to MI. Atg13and LC3II levels were detected by immublot. The densitometric analysis of the bands of three independent experiments are shown. Data are presented as mean±s.e.m. and analysis was performed with one-way ANOVA followed by Tukey–Kramer post hoc analysis. *$P<0.05$; **$P<0.01$.

Supplementary Figure 5 Atg13 interacts with FADD to activate caspase-8 under hypoxia. (a) H9c2 cells were treated with 10μM histamine and with 1μM pyrilamine under hypoxia for 24h. The activation of caspase-8 and caspase-3 was determined by immunoblot analysis. The cleaved caspase-8 was quantified as the percentage of total caspase-8. Results are representative of three independent experiments. (b) H9c2 cells

were infected with adenoviral Atg13 (Ad-Atg13) or were transfected with miR-206//216b antagomir mixture (miR-206//216b Anta) for 12h prior to 10μM histamine treatment under hypoxia for another 12h. The activation of caspase-8 and caspase-3 was determined by immunoblot analyses. The cleaved caspase-8 was quantified as the percentage of total caspase-8. Results are representative of three independent experiments. (c) H9c2 cells were treated with 10μM histamine and 2mM 3-methyladenine (3-MA) under hypoxia for 24h. The activation of caspase-8 and the formation of LC3II were determined by immunoblot. The cleaved caspase-8 was quantified as the percentage of total caspase-8. Results are representative of three independent experiments. (d) H9c2 cells were treated with 110 nM Atg13 siRNA (Atg13-Si) or its negative controls (scramble) for 12h prior to 10μM histamine treatment for 24h. The activation of caspase-8 and the level of Atg13 were determined by immunoblot. The cleaved caspase-8 was quantified as the percentage of total caspase-8. Results are representative of three independent experiments. (e) H9c2 cells were treated with 10μM histamine and 1μM pyrilamine under hypoxia for 24h. The level of FADD was determined by immunoblot. Results are representative of three independent experiments. (f) H9c2 cells were infected with adenoviral Atg13 (Ad-Atg13) or β-gal (Ad-β-gal) and then treated with 10μM histamine under hypoxia for 24h. Immunoprecipitation was performed using an IgG antibody. The levels of Atg13, FADD, pro-caspase and cleaved-caspase-3 were analyzed by immunoblot. Results are representative of three independent experiments. (g) H9c2 cells were infected with adenoviral Atg13 (Ad-Atg13) for 8h prior to 2mM 3-MA treatment

under hypoxia for 24h. Immunoprecipitation was performed using anti-IgG. The levels of Atg13, FADD and pro-caspase were analyzed by immunoblot. Results are representative of three independent experiments. (h) H9c2 cells were infected with adenoviral Atg13 (Ad-Atg13) accompanied by 90nM FADD-Si or its negative controls. Then cells were treated with 10μM histamine under hypoxia for 24h. Immunoprecipitation was performed using an IgG antibody. The levels of Atg13, FADD and pro-caspase were analyzed by immunoblot. Results are representative of three independent experiments. (i) H9c2 cells were transfected with 90 nM FADD-Si or its negative controls. Then cells were treated with 10μM histamine under hypoxia for 24h. The activation of caspase-8 and the level of FADD were determined by immunoblot. The cleaved caspase-8 was quantified as the percentage of total caspase-8. Results are representative of three independent experiments. (j) H9c2 cells were infected with adenoviral Atg13 (Ad-Atg13) accompanied by 90nM FADD siRNA (FADD-Si) or its negative controls. Then cells were treated with 10μM histamine under hypoxia for 24h. Quantitative analysis of apoptosis detected by TUNEL assay is shown. Results are representative of three independent experiments. (k and l) $HDC^{-/-}$ mice were treated with Beclin 1 siRNA (Ad-si-Beclin), Atg13 siRNA (Ad-si-Atg13) adenoviruses or Z-VAD-fmk as described in methods along with 4mg/kg/d histamine, and then subjected to MI. The levels of Atg13, Beclin1, LC3II and p62 were detected 3 days post MI surgery by western blotting (k). The densitometric analysis of the bands of three independent experiments are shown (l). (m) $HDC^{-/-}$ mice were treated as (k). Myocardial cell apoptosis was analyzed by

TUNEL assay. TUNEL-positive myocyte nuclei (apoptotic cells) are green. Nuclei stained by DAPI show blue. Cardiomyocytes were labeled with a-actinin. scale bar, 50 μm. n=3 for the sham group and n=3 for each of the MI groups. Results are representative of three independent experiments. (n) $HDC^{-/-}$ mice were treated as (k). Cardiac function 1 week after MI surgery was analyzed by echocardiographic analysis. LVEF, left ventricular eject fraction. LVFS: left ventricular fraction shortening. n=5 for the sham group and n=5 for each of the MI groups. Data are presented as mean±s.e.m. and analysis was performed with one-way ANOVA followed by Tukey–Kramer post hoc analysis. *P<0.05; **P<0.01.
