# Supplementary material for: Histamine deficiency aggravates cardiac injury through miR-206/216b-Atg13 axis-mediated autophagic-dependant apoptosis
Source: Cell Death Dis. 2018 Jun 7;9(6):694. doi: 10.1038/s41419-018-0723-6 (PMC5992227; doi:10.1038/s41419-018-0723-6)
Supplement: Supplementary file 3 — sumpplementary table 1 [file 41419_2018_723_MOESM3_ESM.pdf]

Supplementary Table 1: miRNAs are potential to function in cardiac autophagy.

miRNAs	Autophagic Targets	Reference
let-7b	caspase-3	1, 2
miR-101	Ras-related protein Rab-5A	3
miR-132	FoxO3 transcription factor	4
miR-133a	unidentified	5
miR-144	unidentified	6
miR-145	fibroblast growth factor receptor substrate 2	7
miR-153	myeloid cell leukemia-1	8
miR-19a-3p	epithelial mesenchymal transition	9
miR-204	LC3-II	10
miR-206	unidentified	11
miR-212	FoxO3 transcription factor	12
miR-21-3p	SH3 domain-containing protein 2	4
miR-216a	Beclin1	13
miR-216b	Beclin1	14, 15, 16, 17
miR-22	p38α	18
miR-221	cyclin-dependent kinase (CDK) inhibitor p27; Ddit4	19, 20, 21
miR-30a	Beclin-1	22, 23, 24
miR-30e	Beclin-1	25
miR-33	unidentified	26
miR-34a	ATG9A	27
miR-365	Skp2	28
miR-451	tuberous sclerosis complex 1	29
miR-497	unidentified	30
miR-99a	unidentified	31

Reference:

1. Ham O, et al. let-7b suppresses apoptosis and autophagy of human mesenchymal stem cells transplanted into ischemia/reperfusion injured heart 7by targeting caspase-3. *Stem Cell Research & Therapy* 2015, **6**(1)**:** 1-11.

2. Cheng J, Zhang P, Jiang H. Let-7b-mediated pro-survival of transplanted mesenchymal stem cells for cardiac regeneration. *Stem Cell Research & Therapy* 2015, **6**(1)**:** 1-3.

3. Wu D, Jiang H, Chen S, Zhang H. Inhibition of microRNA-101 attenuates hypoxia/reoxygenation‑induced apoptosis through induction of autophagy in H9c2 cardiomyocytes. *Molecular Medicine Reports* 2015, **11**(5)**:** 3988.

4. A U, et al. The miRNA-212/132 family regulates both cardiac hypertrophy and cardiomyocyte autophagy. *Nature Communications* 2012, **3**(3)**:** 1078.

5. Nandi SS, et al. Induction of autophagy markers is associated with attenuation of miR-133a in diabetic heart failure patients undergoing mechanical unloading. *American Journal of Translational Research* 2015, **7**(4)**:** 683-696.

6. Li J RS, et al. MicroRNA-144 is a circulating effector of remote ischemic preconditioning. *Basic Res Cardiol* 2014, **109(5):423.**

7. Kenshi H, et al. MicroRNA-145 repairs infarcted myocardium by accelerating cardiomyocyte autophagy. *American Journal of Physiology Heart & Circulatory Physiology* 2015, **309**(11)**:** 1813-1826.

8. Zou Y, Liu W, Zhang J, Xiang D. miR-153 regulates apoptosis and autophagy of cardiomyocytes by targeting Mcl-1. *Molecular Medicine Reports* 2016, **14**(1)**:** 1033.

9. Zou M, et al. Autophagy inhibition of hsa-miR-19a-3p/19b-3p by targeting TGF-β R II during TGF-β1-induced fibrogenesis in human cardiac fibroblasts. *Scientific Reports* 2016, **6:** 24747.

10. Xiao J, et al. MiR-204 regulates cardiomyocyte autophagy induced by ischemia-reperfusion through LC3-II. *Journal of Biomedical Science* 2011, **18**(1)**:** 35.

11. Yang Y, et al. miR-206 Mediates YAP-Induced Cardiac Hypertrophy and Survival. *Circulation Research* 2015, **117**(10)**:** 891.

12. Lin SH, et al. Heme oxygenase-1 modulates microRNA expression in cultured astroglia: implications for chronic brain disorders. *Glia* 2015, **63**(7)**:** 1270-1284.

13. Hui W, et al. miR-21-3p controls sepsis-associated cardiac dysfunction via regulating SORBS2. *Journal of Molecular & Cellular Cardiology* 2016, **94:** 43-53.

14. Menghini R, et al. MiR-216a: a link between endothelial dysfunction and autophagy. *Cell Death & Disease* 2014, **5**(1)**:** e1029.

15. Xu X, et al. MicroRNA-216b/Beclin 1 axis regulates autophagy and apoptosis in human Tenon's capsule fibroblasts upon hydroxycamptothecin exposure. *Experimental Eye Research* 2014, **123:** 43-55.

16. Vacchi-Suzzi C, et al. Perturbation of microRNAs in rat heart during chronic doxorubicin treatment. *Plos One* 2012, **7**(7)**:** e40395.

17. Yuan P, et al. The HIF-2α-MALAT1-miR-216b axis regulates multi-drug resistance of hepatocellular carcinoma cells via modulating autophagy. *Biochem Biophys Res Commun* 2016, **478**(3)**:** 1067-1073.

18. Kan C, Shi W. Autophagy regulates resistance of non-small cell lung cancer cells to paclitaxel.

Tumor Biology 2016, **37**(8)**:** 1-6.

19. G L, et al. miR-22 regulates starvation-induced autophagy and apoptosis in cardiomyocytes by targeting p38α. *Biochemical & Biophysical Research Communications* 2016, **478**(3)**:** 1165-1172.

20. Su M, et al. MicroRNA-221 inhibits autophagy and promotes heart failure by modulating the p27/CDK2/mTOR axis. *Cell Death & Differentiation* 2015, **22**(6)**:** 986-999.

21. Chen Q, Yue Z, Richards AM, Wang P. Up-regulation of miRNA-221 inhibits hypoxia/reoxygenation-induced autophagy through the DDIT4/mTORC1 and Tp53inp1/p62 pathways. *Biochemical & Biophysical Research Communications* 2016, **474**(1)**:** 168-174.

22. Huang J, Huang C, Luo Y, Liu S, Chen X. Role of MiR-30a in cardiomyocyte autophagy induced by Angiotensin II. *Journal of Renin-Angiotensin-Aldosterone System* 2015, **16**(1)**:** 1.

23. Yang Y, et al. Exosomal transfer of miR-30a between cardiomyocytes regulates autophagy after hypoxia. *Journal of Molecular Medicine* 2016, **94**(6)**:** 1-14.

24. Pan W, et al. MiR-30-Regulated Autophagy Mediates Angiotensin II-Induced Myocardial Hypertrophy. *Plos One* 2013, **8**(1)**:** e53950.

25. Lai L, et al. MiRNA-30e mediated cardioprotection of ACE2 in rats with Doxorubicin-induced heart failure through inhibiting cardiomyocytes autophagy. *Life Sciences* 2017, **169:** 69.

26. Ouimet M, et al. Mycobacterium tuberculosis induces the miR-33 locus to reprogram autophagy and host lipid metabolism. *Nature Immunology* 2016.

27. Huang J, et al. miR-34a Modulates Angiotensin II-Induced Myocardial Hypertrophy by Direct Inhibition of ATG9A Expression and Autophagic Activity. *Plos One* 2014, **9**(4)**:** e94382.

28. Wu H, Wang Y, Wang X, Li R, Yin D. MicroRNA-365 accelerates cardiac hypertrophy by inhibiting autophagy via the modulation of Skp2 expression. *Biochemical & Biophysical Research Communications* 2017, **484**(2)**:** 304-310.

29. Song L, et al. MiR-451 is decreased in hypertrophic cardiomyopathy and regulates autophagy by targeting TSC1. *Journal of Cellular & Molecular Medicine* 2014, **18**(11)**:** 2266-2274.

30. Li X, et al. Inhibition of microRNA-497 ameliorates anoxia/reoxygenation injury in cardiomyocytes by suppressing cell apoptosis and enhancing autophagy. *Oncotarget* 2015, **6**(22)**:** 18829-18844.

31. Li Q, et al. Overexpression of microRNA-99a attenuates heart remodelling and improves cardiac performance after myocardial infarction. *Journal of Cellular & Molecular Medicine*

2014, **18**(5)**:** 919-928.
